# Autism Spectrum Disorder and Epilepsy: Pathogenetic Mechanisms and Therapeutic Implications

**DOI:** 10.3390/jcm14072431

**Published:** 2025-04-02

**Authors:** Alessandra Giliberti, Adele Maria Frisina, Stefania Giustiniano, Ylenia Carbonaro, Michele Roccella, Rosaria Nardello

**Affiliations:** 1 Department of Health Promotion, Mother and Child Care, Internal Medicine and Medical Specialities “G. D’Alessandro”, University of Palermo, 90128 Palermo, Italyrosaria.nardello@unipa.it (R.N.); 2 Department of Psychology, Educational Science and Human Movement, University of Palermo, 90128 Palermo, Italy

**Keywords:** autism spectrum disorder—ASD, epilepsy, antiepileptic medication, AED—antiepileptic drugs, DEE—developmental and epileptic encephalopathies, epileptic syndrome

## Abstract

The co-occurrence of autism spectrum disorder (ASD) and epilepsy is a complex neurological condition that presents significant challenges for both patients and clinicians. ASD is a group of complex developmental disorders characterized by the following: (1) Social communication difficulties: challenges in understanding and responding to social cues, initiating and maintaining conversations, and developing and maintaining relationships. (2) Repetitive behaviors: engaging in repetitive actions, such as hand-flapping, rocking, or lining up objects. (3) Restricted interests: focusing intensely on specific topics or activities, often to the exclusion of other interests. (4) Sensory sensitivities: over- or under-sensitivity to sensory input, such as sounds, touch, tastes, smells, or sights. These challenges can significantly impact individuals’ daily lives and require specialized support and interventions. Early diagnosis and intervention can significantly improve the quality of life for individuals with ASD and their families. Epilepsy is a chronic brain disorder characterized by recurrent unprovoked (≥2) seizures that occur >24 h apart. Single seizures are not considered epileptic seizures. Epilepsy is often idiopathic, but various brain disorders, such as malformations, strokes, and tumors, can cause symptomatic epilepsy. While these two conditions were once considered distinct, growing evidence suggests a substantial overlap in their underlying neurobiology. The prevalence of epilepsy in individuals with ASD is significantly higher than in the general population. This review will explore the epidemiology of this comorbidity, delve into the potential mechanisms linking ASD and epilepsy, and discuss the implications for diagnosis, treatment, and management.

## 1. Autism Spectrum Disorder

### 1.1. ASD: General Features and Epidemiology

ASD encompasses a range of neurodevelopmental disabilities marked by repetitive behaviors, specific interests, and challenges in social interactions. It is a complex condition characterized by behavioral and psychological difficulties in children, who often experience distress when their environment changes due to limited adaptive skills. Symptoms typically emerge in early childhood and can significantly impact daily functioning. Additionally, children with ASD are more likely to experience co-occurring language difficulties, intellectual disabilities, and epilepsy compared to the general population. The cause of ASD remains unidentified. The onset can vary widely, and the progression is gradual, lacking a clearly defined starting point [1]. An estimated 61.8 million individuals (one in every 127 people) were on the autism spectrum globally in 2021. The global age-standardized prevalence was 788.3 per 100,000 people, equivalent to 1064.7 autistic males per 100,000 males and 508.1 autistic females per 100,000 females. ASD was ranked within the top ten causes of non-fatal health burden for people younger than 20 years [2].

### 1.2. Diagnostic Procedures

Generally, diagnosing ASD requires a lengthy and time-consuming process that requires a qualified multi-disciplinary team (MDT) composed of a pediatrician, child neuropsychiatrists, psychologists, and speech and occupational therapists. The evaluation of the child usually includes the following:

- A detailed anamnesis to collect information regarding pre- and perinatal personal histories, including prenatal exposure to substances and toxins, birth records, newborn screening results, growth curve analyses, developmental and behavioral histories, learning or psychological difficulties, pharmacological treatment histories, and sleep and food analyses [3];
- A physical examination composed of a growth analysis; general health screening; and neurological examination, including oculomotor behaviors, signs of injury, morphological analyses, and skin examinations [3];
- Hearing screening tests and complete hearing evaluations to rule out the presence of audition impairment [3];
- Genetic testing to identify any underlying genetic conditions associated with ASD;
- Routine biological testing such as complete blood count, urea and electrolytes/glucose, liver function test, thyroid function test, heavy metal levels, HIV testing, and urine screening for aminoaciduria;
- Neuroimaging studies (MRI or CT scan), especially in the presence of suspect metabolic syndrome and to rule out organic causes;
- Electroencephalogram (EEG), especially in the presence of seizures and clinical concerns [3];
- An extensive observation in various settings to assess social interactions, communication skills, and repetitive behaviors;
- Standardized assessment tools, such as the following:
  ○ Cognitive testing, used to assess intellectual functioning for differential diagnosis with other developmental disorders;
  ○ Speech and language evaluation, used to evaluate communication abilities and to determine if there are delays or atypical patterns;
  ○ Behavioral assessments, used to provide a broader perspective on the child’s behavior across different settings;
- Specific diagnostic instruments, such as the following:
  ○ ADOS (Autism Diagnostic Observation Schedule), a semi-structured assessment to evaluate social and communication behaviors;
  ○ Autism Diagnostic Interview—Revised (ADI-R), a structured interview with parents that covers the child’s developmental history and current behaviors.

The diagnosis of ASD is typically made based on the criteria of the *DSM-5TR*, which outline specific requirements related to social communication difficulties and restricted or repetitive behaviors.

### 1.3. Diagnostic Criteria of ASD in DSM-5TR

In the *DSM-5TR* [4], the diagnostic criteria for ASD are as follows (Table 1):

(A) Persistent deficits in social communication and social interaction across multiple contexts, as manifested by all of the following, currently or by history:
  1. Deficits in social-emotional reciprocity, including, for example, abnormal social approach and failure of normal back-and-forth conversation; reduced sharing of interests, emotions, or affect; and failure to initiate or respond to social interactions.
  2. Deficits in nonverbal communicative behaviors used for social interaction, including, for example, poorly integrated verbal and nonverbal communication; abnormalities in eye contact and body language or deficits in understanding and use of gestures; and a total lack of facial expressions and nonverbal communication.
  3. Deficits in developing, maintaining, and understanding relationships, including, for example, difficulties adjusting behavior to suit various social contexts; difficulties in sharing imaginative play or in making friends; and absence of interest in peers.
(B) Restricted, repetitive patterns of behavior, interests, or activities, as manifested by at least two of the following, currently or by history:
  1. Stereotyped or repetitive motor movements, use of objects, or speech (e.g., simple motor stereotypies, lining up toys or flipping objects, echolalia, idiosyncratic phrases).
  2. Insistence on sameness, inflexible adherence to routines, or ritualized patterns of verbal or nonverbal behavior (e.g., extreme distress at small changes, difficulties with transitions, rigid thinking patterns, greeting rituals, need to take the same route or eat the same food every day).
  3. Highly restricted, fixated interests that are abnormal in intensity or focus (e.g., strong attachment to or preoccupation with unusual objects, excessively circumscribed or perseverative interests).
  4. Hyper- or hyporeactivity to sensory input or unusual interest in sensory aspects of the environment (e.g., apparent indifference to pain/temperature, adverse response to specific sounds or textures, excessive smelling or touching of objects, visual fascination with lights or movement).
(C) Symptoms must be present in the early developmental period (but may not become fully manifest until social demands exceed limited capacities or may be masked by learned strategies in later life).
(D) Symptoms cause clinically significant impairment in social, occupational, or other important areas of current functioning.
(E) These disturbances are not better explained by intellectual developmental disorder (intellectual disability) or global developmental delay. Intellectual developmental disorder and ASD frequently co-occur; to make comorbid diagnoses of ASD and intellectual developmental disorder, social communication should be below that expected for general developmental level [4].

Individuals with a well-established DSM-IV diagnosis of autistic disorder, Asperger’s disorder, or pervasive developmental disorder not otherwise specified should be given the diagnosis of ASD. Individuals who have marked deficits in social communication but whose symptoms do not otherwise meet criteria for ASD should be evaluated for social (pragmatic) communication disorder.

The severity of the social communication impairments and of the restricted, repetitive patterns of behavior must be specified as follows:

(1) Level 3—Requiring very substantial support: The patient presents with severe deficits in verbal and nonverbal social communication skills, which cause severe impairments in functioning, very limited initiation of social interactions, and minimal response to social overtures from others, inflexibility of behavior, extreme difficulty coping with change, or other restricted/repetitive behaviors that markedly interfere with functioning in all spheres. Changes in their routine usually cause great difficulty and distress.
(2) Level 2—Requiring substantial support: The patient presents with marked deficits in verbal and nonverbal social communication skills; social impairments apparent even with supports in place; limited initiation of social interactions; and reduced or abnormal responses to social overtures from others. The patient presents with distress and/or difficulty changing focus or action.
(3) Level 1—Requiring support: The patient presents, without supports in place, with deficits in social communication that cause noticeable impairments. He has difficulties initiating social interactions and has clear examples of atypical or unsuccessful responses to social overtures of others. He may appear to have decreased interest in social interactions. Inflexibility of behavior causes significant interference with functioning in one or more contexts. The patient also has difficulties switching between activities, and problems related to organization and planning hamper independence.

It must be specified if the child also presents, in compresence with the ASD, intellectual impairment; language impairment; a known genetic or other medical condition; a neurodevelopmental, mental, or behavioral disorder; or catatonia [4].

ASD is characterized by challenges in reciprocal social communication and social interaction (Criterion A), along with restricted and repetitive patterns of behavior, interests, or activities (Criterion B). These symptoms typically manifest during early childhood and can significantly impact the daily functioning of those children (Criteria C and D). The age at which functional impairments becomes evident differs based on the individual’s characteristics and their environment. While core diagnostic features are apparent during the developmental period, interventions, compensatory strategies, and existing support systems can sometimes obscure these challenges in certain contexts. The expression of ASD varies a lot, influenced by factors such as the severity of the condition, developmental level, and chronological age. Individuals without cognitive or language impairments may exhibit more subtle deficits in social communication (Criterion A) and restricted interests or behaviors (Criterion B) than patients with intellectual disability or language delay. This can lead to significant efforts to mask these deficits, particularly in individuals with stronger overall communication skills. Deficits in social communication may appear less pronounced in those who are verbally fluent and do not have intellectual impairments. Similarly, restricted interests may be more aligned with age-appropriate norms, making them less noticeable. ASD encompasses early infantile autism, Kanner’s autism, high-functioning autism, atypical autism, pervasive developmental disorder not otherwise specified, childhood disintegrative disorder, and Asperger’s disorder. The impairments identified in Criterion A are pervasive and long-lasting. Diagnoses are most reliable when informed by multiple sources, including clinician observations (ADOS), caregiver reports, and, where possible, self-reports. Manifestations of verbal and nonverbal deficits in social communication can be based on the individual’s age, intellectual ability, and language skills, as well as their treatment history and current support. Many individuals with ASD experience language difficulties, which can range from total lack of speech to language delays, poor comprehension, echolalia, or rigid and overly literal language use. Even when formal language abilities (e.g., vocabulary and grammar) are intact, the application of language in reciprocal social communication is often impaired. Deficits in social-emotional reciprocity may be evident in young children who show limited or no initiation of social interactions, lack of emotional sharing, and reduced imitation of others’ behaviors. When communication does occur, it is frequently one-sided, focusing on requests or labeling rather than engaging in mutual conversation or emotional exchange.

In older children and adults without intellectual disabilities or language delays, challenges in social-emotional reciprocity are usually most noticeable in their difficulties in interpreting and responding to complex social cues. Even those who have developed compensatory strategies for social interactions may struggle in unfamiliar or unsupported situations, leading to significant mental effort and anxiety as they navigate social conventions. This can contribute to an underrecognition of ASD, particularly among adult women. Comprehensive assessments, including extended observation in naturalistic settings and inquiries about the emotional toll of social interactions, may be essential to diagnose ASD [4].

### 1.4. Clinical Features

ASD is characterized by deficits in nonverbal communicative behaviors essential in the child for social interaction. These deficits may manifest as absent, reduced, or atypical use of eye contact, lack of gestures (indicative pointing or crossmodal gestures), inadequate modulation of facial expressions, body orientation, or variations in speech intonation (vocal tone anomalies or monotony) often deviating from cultural norms. A hallmark of ASD is impaired joint attention, which can present as a lack of pointing, showing, or bringing objects to share interests with others, as well as difficulty in following another person’s pointing or gaze. While some individuals may acquire a limited set of functional gestures, their overall repertoire tends to be smaller compared to their peers, and they often do not use expressive gestures spontaneously during communication. Among youth and adults with fluent language skills, the coordination of nonverbal cues with verbal communication can appear awkward or exaggerated, resulting in a perception of “odd” or “wooden” body language. Although deficits may be subtle in specific areas—such as maintaining eye contact while speaking—there is often a noticeable lack of integration among eye contact, gestures, posture, prosody, and facial expressions in social contexts. The impairment can become more pronounced under stress or during prolonged interactions. When assessing the ability to develop, maintain, and understand relationships in individuals with ASD, it is essential to consider age, gender, and cultural norms. Individuals may display absent, reduced, or atypical social interest, which can manifest as rejection of others, passivity, or inappropriate approaches that may seem aggressive or disruptive. These challenges are particularly evident in young children, who may lack shared social play and imaginative interactions typical of their developmental stage, often opting for rigid play structures instead. As these individuals grow older, they may present with difficulties in understanding and navigating the nuances of social behavior—failing to recognize what is deemed appropriate in various contexts, such as the difference between casual behavior during a job interview and during informal interactions. Furthermore, they may prefer isolating themselves and show a preference for solitary activities or on the other hand seek interactions with individuals significantly younger or older than themselves. While there is often a desire to form friendships, this may occur without a complete understanding of the dynamics of friendship, leading to one-sided relationships or connections based solely on shared special interests. ASD is further defined by restricted, repetitive patterns of behavior, interests, or activities, as outlined in diagnostic criteria. These behaviors can, in their manifestations, be influenced by factors such as age, cognitive ability, intervention, and available support. Individuals may exhibit simple motor stereotypies such as hand flapping, finger flicking, repetitive use of objects such as spinning coins, lining up toys, or repetitive speech patterns such as echolalia or delayed or immediate repetition of heard phrases. A strong adherence to routines may be observed, resulting in distress at seemingly minor changes or an insistence on following rigid rules. Individuals may engage in ritualized verbal or nonverbal behaviors, such as repetitive questioning or pacing defined areas. Highly restricted interests are another characteristic of ASD. Sensory sensitivities can also play a role in ASD, with some individuals displaying extreme reactions to certain sounds or textures, a fascination with lights or spinning objects, and even a notable indifference to physical discomfort. Although many individuals with ASD who do not have intellectual or language impairments may learn to suppress their repetitive behaviors in public settings, these behaviors often serve a calming or self-soothing purpose. Special interests can provide significant joy and motivation.

Criterion D stipulates that the features of this disorder must lead to clinically significant impairments in social, occupational, or other vital areas of functioning. Criterion E emphasizes that while social communication deficits may co-occur with intellectual developmental disorder (intellectual disability), these impairments must be disproportionate to the individual’s developmental level, indicating that difficulties extend beyond what is typically expected based on development [4].

### 1.5. Associated Features

One significant aspect of ASD is the frequent co-occurrence of intellectual (33%) and language impairments (16%). Many individuals with ASD exhibit delays in language development and typically have smaller vocabularies than their peers, as shown by the Mullen Scales of Early Learning (MSEL) and the MacArthur–Bates Communicative Developmental Inventory (CDI) [5,6]. More often, they demonstrate comprehension skills that lag behind their expressive abilities. Even those with average or above-average intelligence often present with an uneven profile of cognitive strengths and weaknesses, leading to a notable disparity between their intellectual capabilities and adaptive functional skills. A common feature of ASD is the presence of theory-of-mind deficits, which manifest as difficulties in understanding and interpreting the perspectives of others. Additionally, executive function deficits are prevalent among individuals with ASD, further complicating their ability to plan, organize, and regulate their behaviors. Challenges with central coherence—an ability to grasp context and perceive the overarching significance of information—are also frequently observed, leading to a tendency to focus intensely on details at the expense of the broader picture. Motor skills can also be affected in individuals with autism. Many exhibit atypical motor patterns, such as an unusual gait or clumsiness, or other abnormal motor behaviors, including walking on tiptoes [5]. The main motor domains evaluated include features of fine and gross motor skills, manual dexterity, coordination/motor control/praxis, balance, running speed/agility, strength, gait, whole-body movements, aiming and catching (ball skills), and repetitive movements. Motor impairments are consistently observed in ASD from the first years of life, persisting into adulthood. They include a significant deficit in performance of manual, posture, strength, and gait behavior/skills. The deficits described in ASD comprise impairments in fine and gross motor skills, lower balance, lower cadence, and greater variation of gait control, as well as weakness among other features that lead this population to move in an adaptive way, affecting their interactions in real life [7]. These motor deficits can be accompanied by self-injurious behaviors, such as head banging or wrist biting, which are more prevalent in children and adolescents with ASD compared to those with other developmental disorders, including intellectual disability. In some cases, individuals with autism may display catatonic-like motor behaviors, with episodes of slowing or “freezing” mid-action, with marked deterioration in motor symptoms, mutism, posturing, grimacing, and waxy flexibility, especially during adolescent years. In a study of about 48 autistic patients, (11 female, 37 male) aged 12–18, the presence of catatonia was detected in 18 out of 48 (37.5%) participants according to BCRS and in 16 cases (35.4%) according to DSM-5 criteria [8].

### 1.6. Epidemiology

ASD represents an important public health concern. The World Health Organization (WHO) estimates the international prevalence of ASD at approximately 0.76% (only about 16% of the global child population). In the United States, the Centers for Disease Control and Prevention (CDC) reports a higher prevalence of around 1.68% among children aged 8 years. ASD affects individuals of all racial, ethnic, and socioeconomic backgrounds; however, disparities in diagnosis persist. Caucasian children are consistently identified with ASD at higher rates than their Black or Hispanic counterparts, although this gap may be influenced by factors such as stigma, limited access to healthcare services, and language barriers for non-English speaking patients. The male-to-female ratio for ASD diagnoses has traditionally been reported as 4:1, but recent meta-analyses suggest a more accurate ratio closer to 3:1. (ASD is more common in groups with Y chromosome aneuploidy. Males with Y chromosome aneuploidy—XYY and XXYY—were 4.8 times more likely to have a diagnosis of ASD than the XXY/KS—Klinefelter syndrome—group and 20 times more likely than males in the general population. Y-chromosome genes may provide insight into male predominance in idiopathic ASD [9,10,11].) This discrepancy may also be attributed to underdiagnosis in females, who often exhibit less overt symptoms and may engage in “camouflaging” behaviors to mask social deficits. Females with autism tend to have more severe intellectual disability [12]. Certain genetic conditions are frequently associated with a higher incidence of co-occurring ASD, including:

- Fragile X syndrome (first described by Martin and Bell in 1943, a common monogenic cause of syndromic ASD, characterized by cognitive disability, delayed or absent speech, autistic features, attention deficit, hyperactivity, anxiety, a high incidence of epilepsy, and macroorchidism and estimated to be about 2% of all ASD cases) [13];
- Tuberous sclerosis (an autosomal dominant genetic disorder affecting ~1 in 6000 live births, characterized by benign tumors in the brain and other organs, epilepsy, cognitive impairment, and high penetrance of ASD, caused by mutations in the *TSC1* or *TSC2* genes, which encode hamartin and tuberin, that dimerize and form a complex that negatively regulates the mammalian target of rapamycin (mTOR) protein complex). The ASD prevalence is estimated to be between 36% and 50% [13];
- Down syndrome, a trisomy of chromosome 21, characterized by distinct facial features, such as a flat facial profile, slanted eyes, and a short neck, single transverse palmar crease, a gap between the first and second toes, and hypotonia, mild to moderate intellectual disability, congenital heart defects, respiratory issues, hearing problems, and thyroid conditions. The ASD prevalence is estimated to be between 5% and 39% [14];
- Rett syndrome, a progressive neurological disorder usually caused by loss-of-function mutations in the methyl-CpG-binding protein 2 (*MECP2*) gene, which affects ~1 in 10,000 female live births and is characterized by 6–18 months of normal development followed by rapid regression, autistic features, loss of purposeful hand use and language skills, cognitive deficits, motor impairments, breathing abnormalities, seizures, and acquired microcephaly [13];

However, these genetic disorders account for a relatively small proportion of overall ASD cases. Advances in genetic testing, particularly chromosomal microarray analysis, have linked several chromosomal sites (X, 2, 3, 7, 15, 16, 17, and 22) to increased ASD risk [15]. In addition to genetic factors, environmental influences, such as advanced parental age and prematurity, have been identified as risk factors for ASD. Firstborn offspring of two older parents were three times more likely to develop autism than were third- or later-born offspring of mothers aged 20–34 years and fathers aged <40 years [16]. The association between older parental age and ASD may be related to the increased probability of genetic mutations in older gametes, which can contribute to obstetric complications, including prematurity [9]. Others factors, such as White and Asian race, gestational hypertension, gestational diabetes, maternal and paternal education college graduate, threatened abortion, antepartum hemorrhage, caesarian delivery, gestational age ≤36 weeks, parity ≥4, spontaneous labor, induced labor, no labor, breech presentation, preeclampsia, fetal distress, low birth weight, postpartum hemorrhage, male gender, and brain anomaly, increase the risk of incidence of ASD [17].

### 1.7. Development and Course

ASD is characterized by behavioral features that usually emerge in early childhood. Some children may exhibit a lack of interest in social interactions during their first year of life, while most symptoms are recognized between 12 and 24 months of age. In cases of notable developmental delays, symptoms may present earlier than 12 months, and in instances where behaviors are subtler, recognition may occur later than 24 months. The onset of ASD often includes descriptions of early developmental delays or the loss of previously acquired social or language skills (regression). Parents or caregivers may report a gradual or rapid decline in social behaviors or communication abilities, particularly between 12 and 24 months of life of the child. Prospective studies indicate that the onset of ASD is frequently associated with a decline in critical social and communication behaviors within the first two years of life. Such declines are uncommon in other neurodevelopmental disorders, making them an important indicator of ASD. In rare instances, developmental regression occurs after at least two years of typical development (previously categorized as childhood disintegrative disorder in the *DSM-IV*). The initial symptoms of ASD often involve delayed language development, limited social interest, or atypical social interactions. Other behaviors may include unusual play patterns, like carrying toys without engaging with them, and atypical communication habits. As children progress into their second year, odd and repetitive behaviors become more pronounced, along with a noticeable absence of typical play. Differentiating between restrictive and repetitive behaviors indicative of ASD and those typical of typically developing children can be challenging, especially among preschoolers. The clinical distinction relies on the type, frequency, and intensity of these behaviors. Learning and compensatory strategies can develop throughout an individual’s life. Symptoms often peak in early childhood and early school years, with many children demonstrating developmental gains in later childhood, particularly in social interaction. Although a small percentage of individuals may experience behavioral decline during adolescence, most show improvement. Generally, individuals with lower levels of impairment tend to be more independent. However, even those with milder symptoms may present difficulties with social naivety, vulnerability, and difficulties in managing practical demands without support, often experiencing anxiety and depression. Many adults with ASD report employing compensation strategies and coping mechanisms to mask their challenges in social settings, which can lead to significant stress. Diagnosis in adulthood can arise from various triggers, including familial diagnoses or personal crises. In such cases, obtaining a developmental history may be difficult, but a diagnosis can still be made based on current criteria and supporting childhood history. Overall, ASD symptoms can persist and cause significant impairments in key life areas, even if interventions or coping strategies help mask these challenges [4].

### 1.8. Etiology and Pathogenesis

ASD is a complex neurobiological condition influenced by both genetic and environmental factors that affect brain development. Research on ASD has primarily focused on brain development and function, revealing significant central nervous system (CNS) pathologies at both gross and cellular levels. Studies have noted morphological abnormalities such as increased head circumference and altered brain structures in young children with ASD. Notably, evidence also suggests a pattern of early brain overgrowth followed by growth arrest or degeneration. Patients with ASD often present characteristic alterations in cerebellar architecture and connectivity, abnormalities in the limbic system, and cortical changes in the frontal and temporal lobes, alongside other subtle malformations. The majority of ASD patients exhibit focal disruptions in cortical laminar architecture, suggesting challenges in cortical layer formation and neuronal differentiation. Brain overgrowth—reflected in both increased cortical size and extra-axial fluid—has also been documented in children with ASD, such as altered brain connectivity patterns, with some studies indicating lower connectivity between distant brain regions and increased connectivity within nearby regions. Synaptic dysfunction is identified as a key neuropathology in ASD, with many high-risk genes linked to synaptic function. Specific signaling pathways, such as mTOR/PI3K and NRXN-NLGN-SHANK, are crucial in ASD pathology, affecting synaptogenesis and leading to a potential imbalance in excitatory and inhibitory transmission. Beyond the brain, recent research has highlighted the role of the gastrointestinal (GI) system in ASD. A significant number of individuals with ASD report GI dysfunction, which is often correlated with the severity of ASD symptoms. Increased intestinal permeability, often referred to as “leaky gut,” has been observed, suggesting a connection between gut health and chronic inflammation in ASD. The gut–brain axis is recognized for its influence on behavior and neurodevelopment, with dysbiosis in gut microbiota reported in individuals with ASD, indicating differences in microbial diversity and composition compared to non-autistic individuals. These alterations in gut microbiota and intestinal barrier function may exacerbate GI issues and inflammatory processes, thereby impacting cerebral function and contributing to ASD neuropathology [9,18]. Genetic factors play a significant role in susceptibility to ASD. Studies indicate that siblings of individuals with ASD have a higher risk of diagnosis compared to population norms, and monozygotic twins show a much higher risk of developing autism. (47–96% ASD concordance rates for monozygotic twins and 0–36% in dizygotic twins) [19]. Advances in genetic research, including genome-wide association studies and whole exome sequencing, have expanded our understanding of the genes associated with ASD susceptibility. Candidate genes often involve those linked to brain development, neurotransmitter functioning, or neuronal excitability. Many genetic variants associated with ASD encode proteins crucial for neuronal synapse functionality or are involved in activity-dependent neuronal changes, including regulatory proteins such as transcription factors. Networks of genetic risk may converge on pathways related to neurotransmission and neuroinflammation. Dysregulation of transcription and splicing, along with alterations in epigenetic mechanisms like DNA methylation and histone modification, may further contribute to ASD pathogenesis. Recent studies have identified 16 novel genes associated with ASD, hinting at new mechanisms involving cellular cytoskeletal structure and ion transport. [9] Overall, ASD is characterized by significant genetic heterogeneity, with de novo and inherited variants identified in over 700 genes [15,20,21,22,23,24]. While genetic factors are crucial in ASD etiology, the phenotypic expression of genetic susceptibility is highly variable. Environmental influences during prenatal, perinatal, and postnatal periods may modulate genetic risk in certain individuals [25]. Prenatal exposure to medications such as thalidomide and valproic acid has been associated with an increased risk of ASD, whereas folic acid supplementation during pregnancy may reduce risk, particularly in those exposed to antiepileptic drugs. Maternal autoimmune diseases, maternal infection, or immune activation during pregnancy have been suggested as potential risk factors. In addition, inter-pregnancy intervals that are either too short or too long have also been correlated with increased ASD risk [9].

### 1.9. Association with Suicide Thought

Individuals with ASD are at greater risk for suicide death compared to those without ASD. Children with ASD who had impaired social communication had a higher risk of self-harm with suicidal intent, suicidal thoughts, and suicide plans by age 16 years compared to those without impaired social communication. Adolescents and young adults with ASD have an increased risk of suicide attempts compared to age- and sex-matched control subjects, even after adjustments for demographic factors and psychiatric comorbidities [4]. Self-injurious thoughts and behaviors (SITBs) are prevalent among autistic youth. Existing research predominantly relies on caregiver reports, often overlooking the perspectives of the youth themselves. Critical aspects of SITBs—including the types of behaviors, thought content, triggers, and the decision-making process regarding whether to disclose these thoughts and behaviors to caregivers—remain underexplored. This gap in understanding presents significant challenges for clinicians and families attempting to provide effective support to young people with autism in crisis. In a study conducted by Jessica M Schwartzman and her team, involving 103 autistic patients aged 10 to 17 without intellectual disabilities who were receiving outpatient mental health services. The study used the Columbia-Suicide Severity Rating Scale (C-SSRS) clinical interview and supplemented it with follow-up questions aimed at gaining deeper insight into youths’ perceptions of suicide, the triggers for their suicidal thoughts, and their communication with caregivers regarding these feelings. The results revealed that a substantial majority of participants reported having experienced suicidal thoughts at some point in their lives (86; 83.5%). Among these, specific thoughts such as dying/suicide (20; 23.3%) and death by cutting (13; 15.1%) were notably common. Alarmingly, half of the youth who experienced suicidal thoughts (43; 50.0%) did not disclose these feelings to their caregivers. Furthermore, nearly one-quarter of participants reported having attempted suicide at some point (25; 24.3%), while a smaller group (16; 15.5%) sought assistance from caregivers to avert an attempt. The study identified sadness/depression and bullying/teasing as the predominant triggers for suicidal behaviors, whereas anger/frustration emerged as the primary trigger for non-suicidal self-injury. These findings underscore the necessity of incorporating youth perspectives into the assessment and prevention strategies for SITBs among autistic young people. By enhancing current assessment tools and prevention approaches, caregivers and clinicians can better support autistic young people facing crises [25].

### 1.10. Functional Consequences of ASD

In young children with ASD, deficits in social and communication skills can significantly hinder learning, particularly in social interactions and peer-based settings. At home, strong adherence to routines, aversion to change, and sensory sensitivities can complicate essential activities such as eating and sleeping, making routine care—like haircuts and dental visits—particularly challenging. Typically, adaptive skills in these individuals tend to be lower than their measured IQ levels. Difficulties with planning, organization, and adjustment to change can adversely affect academic performance, even among those with above-average intelligence. As these individuals transition into adulthood, challenges in achieving independence often persist due to ongoing rigidity and difficulty handling novel situations. Many adults with ASD, even those without co-occurring intellectual developmental disorders, experience poor psychosocial functioning, as evidenced by low rates of independent living and gainful employment. The long-term functional consequences of these challenges in older age remain largely unknown; however, it is likely that social isolation and communication difficulties—such as reduced help-seeking behavior—may negatively impact health outcomes in later adulthood. Additionally, individuals with ASD who have co-occurring intellectual developmental disorders, epilepsy, mental health issues, and chronic medical conditions may face an increased risk of premature mortality. Notably, rates of death from injury and poisoning are higher in this population compared to the general public, as are suicide rates. Among children with ASD, drowning remains the leading cause of accidental death [4]. Autistic people have at least a two times higher risk of dying than other people, and females with ASD are at an even greater risk of death compared to males [26].

### 1.11. Differential Diagnoses for ASD

- *Attention Deficits and Hyperactivity*. Individuals with ASD often exhibit either heightened focus, distractibility, or hyperactivity. Attention-deficit/hyperactivity disorder (ADHD) frequently occurs in association with ASD. While both conditions can present attentional difficulties, ADHD is characterized by a developmental trajectory that lacks the restricted, repetitive behaviors typical of ASD.
- *Intellectual Developmental Disorder*. Differentiating intellectual developmental disorder (IDD) from ASD can be particularly challenging in young children, especially when language and symbolic skills are very underdeveloped. ASD is diagnosed when social communication and interaction are significantly impaired compared to the individual’s nonverbal skills. IDD is diagnosed when social communicative skills align with other intellectual abilities.
- *Language Disorders and Social (Pragmatic) Communication Disorder*. Language disorders may include communication difficulties that result in social challenges, but they typically do not involve abnormal nonverbal communication or restricted behaviors. When social communication impairments are present without restricted or repetitive behaviors, a diagnosis of social (pragmatic) communication disorder may be appropriate.
- *Selective Mutism*. Selective mutism presents with appropriate communication skills in certain contexts, and social reciprocity remains intact. Unlike ASD, selective mutism does not involve impaired social interaction or the presence of restricted behaviors.
- *Stereotypic Movement Disorder*. Motor stereotypies are common in ASD, and when these behaviors do not lead to self-injury, a diagnosis of stereotypic movement disorder is generally not warranted. However, if such behaviors result in self-harm and require treatment, both diagnoses may be appropriate.
- *Anxiety Disorders*. Anxiety disorders frequently overlap with ASD symptoms, complicating diagnosis. Common anxiety presentations in individuals with ASD include specific phobias and social anxiety. Clinicians must discern whether behaviors such as social withdrawal stem from anxiety or are intrinsic to ASD.
- *Obsessive-Compulsive Disorder*. Repetitive behaviors are central to both obsessive-compulsive disorder (OCD) and ASD, but their motivations differ. In OCD, compulsions are responses to intrusive thoughts aimed at alleviating anxiety, while in ASD, repetitive behaviors may have a more pleasurable or reinforcing quality.
- *Schizophrenia*. Childhood-onset schizophrenia typically follows a period of normal development, with prodromal symptoms that may resemble those in ASD. Distinct features of schizophrenia, such as hallucinations and delusions, are absent in ASD. However, both disorders can co-occur, and careful assessment is necessary to differentiate between them.
- *Personality Disorders*. In adults with ASD who do not have significant intellectual or language impairments, behaviors may be misinterpreted as symptoms of personality disorders, such as narcissistic or schizotypal personality disorder. The early developmental history of autism, including lack of imaginative play and sensory sensitivities, can aid in distinguishing it from personality disorders [4].

### 1.12. Possible Associations

In the context of ASD, individuals experience a higher incidence of various medical conditions compared to the general population. Research indicates that children with autism present frequently with eczema, asthma, food allergies, gastrointestinal (GI) issues, and other symptoms [27,28,29,30]. For instance, they are 1.6 times more likely to have eczema, 1.8 times more likely to suffer from asthma and food allergies, and up to 7 times more likely to report GI problems [31]. Identifying comorbid conditions in children with autism is crucial. Many of these medical issues can exacerbate the core symptoms of autism—such as social deficits, language impairments, and repetitive behaviors—and may hinder the child’s ability to learn effectively. When these comorbidities are treated, improvements in behavior and learning outcomes can often be observed. Furthermore, the increased mortality risk associated with ASD is likely linked more to these comorbid medical conditions and intellectual disabilities than to autism itself. Therefore, timely recognition and treatment of comorbid conditions can significantly enhance the quality of life for both the child and their family. However, diagnosing comorbid conditions in children with ASD can be challenging due to various factors, including communication difficulties, symptom ambiguity, and the evolving nature of these symptoms over time. The common misconception that certain behaviors are merely part of autism adds another layer of complexity. There is also a lack of diagnostic tools specifically designed to screen for these disorders. Symptoms that may appear to be exclusively related to autism, such as headbanging or fidgeting, could actually indicate underlying medical issues, such as headaches or gastrointestinal discomfort. Recognizing and addressing these comorbidities is essential for ensuring effective treatment and care [32].

- *Genetic disorders*

Genetic disorders are linked to an increased risk of autism. These include Fragile X syndrome (FXS), Down syndrome (DS), Duchenne muscular dystrophy, neurofibromatosis type I (NF1), tuberous sclerosis complex (TSC), and Rett syndrome. The interplay of these genetic factors can lead to atypical brain wiring associated with ASD. FXS is the most common inherited intellectual disability and is present in approximately 2–3% of all ASD cases, with 25–33% of FXS patients also diagnosed with ASD. Children with both FXS and ASD exhibit higher rates of social anxiety, intellectual disabilities, and repetitive behaviors compared to those with ASD without known genetic causes. The co-occurrence of ASD and DS is noteworthy, as up to 40% of children with DS may also have ASD. DS-ASD patients often display developmental regression, communication challenges, and various behavioral issues. Duchenne muscular dystrophy is another condition frequently observed alongside ASD, particularly in children exhibiting toe-walking, necessitating creatine phosphokinase (CPK) level assessments. Neurofibromatosis type I has been associated with ADHD symptoms, though recent studies suggest a lesser prevalence of ASD in this population. TSC presents a significant link to ASD, with prevalence rates ranging from 26% to 45%, as does Rett syndrome, with a prevalence of 61% of children. Given the higher incidence of genetic disorders in children with autism, genetic consultation is advisable for any child diagnosed with ASD. This can help inform treatment pathways and improve developmental outcomes [32].

- *Neurological disorders*

Children with autism are at an increased risk for various neurological disorders, including epilepsy, macrocephaly, hydrocephalus, cerebral palsy, and migraines. The overlap between autism and these neurological conditions suggests shared molecular mechanisms. Up to 60% of children with autism may show abnormal electroencephalogram (EEG) results, and 10% to 30% may develop epilepsy. Conversely, autism occurs in approximately 8% of children with epilepsy. The co-occurrence of epilepsy and autism may result from shared pathogenic mechanisms, including synaptic dysfunction and neuroinflammation. Early seizures can also lead to “autism-like” behaviors. Children with ASD and intellectual disabilities are at an even higher risk for epilepsy, particularly with underlying neurological conditions such as TSC [32,33,34,35,36,37,38].

- *Sleep disorders*

Sleep disorders are prevalent in children with autism, affecting approximately 80% of this population. Common sleep issues include difficulty falling asleep, frequent awakenings, and sleepwalking. These disturbances can significantly impact the child’s behavior, learning, and overall well-being, with potential links to GI disorders and anxiety. Given the treatable nature of many sleep disorders, it is imperative to implement evidence-based interventions to improve sleep quality and, consequently, overall functioning for children with ASD [32,39,40,41,42].

- *GI disorders*

Gastrointestinal (GI) issues are common among children with ASD, with prevalence rates ranging from 46% to 84%. Common manifestations include chronic constipation, diarrhea, and gastroesophageal reflux. These GI symptoms can lead to discomfort, which may exacerbate behavioral issues and hinder learning. The identification of underlying GI disorders is crucial, as untreated conditions can significantly affect the child’s quality of life. Clinical assessments should include thorough GI histories, as many children with autism may struggle to communicate their discomfort effectively [32,43,44,45,46].

- *Metabolic disorders*

Inborn errors of metabolism, such as mitochondrial dysfunction, have been increasingly linked to autism. These disorders can manifest as various neurological symptoms and affect the child’s overall health and development. Laboratory findings, including abnormal blood chemistry and urine analysis, can assist in identifying potential metabolic disorders in children with autism [32,47,48,49].

- *Immune, autoimmune, and allergic disorders*

Research suggests that children with ASD may experience immune dysregulation and chronic neuroinflammation. Approximately 25% of children with autism exhibit immune deficiencies, which can complicate their overall health and treatment outcomes. Identifying and managing these immune dysfunctions can lead to improved cognitive and behavioral outcomes. Allergic conditions are also more prevalent in children with ASD, influencing both the severity of symptoms and behavioral challenges. Addressing allergic disorders can be beneficial for improving the overall functioning of children with autism [32,50,51].

- *Emergency room and outpatient guidelines*

Children with ASDs encounter a heightened risk of medical emergencies, particularly in the emergency department (ED) setting. This risk increases to 70% in teens between the ages of 15 and 18 years. Effective management requires specialized training for ED staff and consideration of the unique challenges faced by children with autism. Recommendations for creating an autism-friendly environment in the ED include reducing sensory stimuli, offering calming objects, and ensuring adequate communication with families [32,52,53,54,55,56].

### 1.13. Pharmacological Treatment for ASD

Refs. [57,58,59,60,61,62,63,64,65,66,67,68,69,70,71,72,73,74,75,76,77,78,79,80,81,82,83,84,85,86,87,88,89,90,91,92,93,94,95,96,97,98,99,100,101,102,103,104,105,106,107,108,109,110,111,112,113,114,115,116,117,118,119,120,121,122,123,124,125,126,127,128,129,130,131,132,133,134,135,136,137,138,139,140,141,142,143,144] In clinical practice, psychopharmacological treatments are frequently used to manage symptoms associated with comorbidities in individuals with ASD [Table 2].

Emotional dysregulation and irritability are prevalent in this population, often manifesting as aggression toward oneself or others, tantrums, and rapidly changing moods. These behaviors can significantly impact overall adaptive functioning in both children and adults. Individuals with ASD often struggle with emotional awareness, emotional language, flexibility, and sensitivity to change and environmental stimuli, making them particularly vulnerable to emotional dysregulation. Emotional dysregulation is defined as the ability to manage arousal and emotional reactivity to achieve goals and maintain adaptive behaviors. Research indicates that as many as 68% of individuals with ASD may exhibit aggression or self-injurious behaviors (SIB), based on parent-reported measures, though more objective assessments suggest a lower prevalence. The presence of irritability and aggression can lead to serious personal and social consequences, including significant functional impairment, increased family stress, and the need for residential placement.

Pharmacological treatments for irritability and aggression in individuals with ASD typically include antipsychotics, mood stabilizers, and glutamatergic blockers. Currently, only two medications are approved in the US and many European countries specifically for treating these symptoms in individuals with ASD: aripiprazole and risperidone, which are both indicated for children and adolescents. The most common side effects associated with aripiprazole include akathisia, while risperidone is often linked to weight gain. Reports indicate that adverse effects occur in up to 61% of patients treated with aripiprazole and 77% of patients treated with risperidone. Other atypical antipsychotics, including quetiapine, ziprasidone, and paliperidone, may be used for patients who do not respond to initial treatments. Olanzapine is less commonly prescribed due to its high risk of causing weight gain. Clozapine can be considered for treatment-resistant patients displaying significant disruptive behaviors, but its use is limited by potential risks such as agranulocytosis and myocarditis. First-generation antipsychotics, like haloperidol and chlorpromazine, are also options for treatment-resistant irritability or aggression, although careful monitoring for extrapyramidal side effects is necessary. Valproate serves as an alternative therapy, particularly in patients with concurrent epilepsy, for addressing irritability. N-acetylcysteine (NAC) has been associated with a notable decrease in hyperactivity and irritability. For patients with co-occurring ADHD, methylphenidate and alpha-2 adrenergic agonists are recommended to manage hyperactivity and attention difficulties. Methylphenidate, a psychostimulant, is the first medication widely utilized for ADHD treatment. While its effectiveness in children with ASD may not match that seen in typically developing children, it remains a reasonable first-line option for untreated children with ASD and uncomplicated ADHD. Children with autism may experience a higher incidence of side effects, including decreased appetite, sleep issues, irritability, headaches, and stomach discomfort. Clonidine and guanfacine, which act on α2-adrenergic presynaptic receptors to inhibit noradrenergic release and synaptic transmission, have demonstrated significant improvements in ADHD symptoms, as reported by parents and teachers. To manage tics in patients with both ASD and Tourette syndrome, some literature suggests efficacy for topiramate and aripiprazole, while levetiracetam has not shown effectiveness. Pharmacological treatment options for stereotypies include risperidone and pentoxifylline. Regarding anxiety disorders, which are commonly comorbid with ASD and affect between 35% and 75% of young people, antidepressants and anxiolytics are often employed, but their effectiveness is generally limited. Selective serotonin reuptake inhibitors (SSRIs) are frequently referenced in literature for treating anxiety and depression in ASD patients, with citalopram, fluoxetine, and sertraline noted for their effectiveness and minimal side effects. Alternatives for anxiety treatment include noradrenergic and specific serotonergic antidepressants (NaSSA) like mirtazapine and buspirone, a serotonin 5-HT1A receptor agonist. Benzodiazepines are commonly prescribed to young people with ASD, particularly for sleep disturbances and acute anxiety. Methylphenidate and risperidone have also been utilized for depression in individuals with co-occurring ASD. Melatonin is a primary pharmacological intervention for sleep disturbances, often combined with behavioral treatment, and has demonstrated effectiveness in enhancing sleep duration. Other pharmacological options such as clonidine and clonazepam are also considered for improving sleep initiation and overall quality. The treatment of obsessive-compulsive disorder (OCD) in patients with ASD includes SSRIs and antipsychotics. Clomipramine has shown some efficacy in small studies for managing repetitive and compulsive behaviors in children and adolescents with ASD, although it is often associated with side effects and is typically regarded as a second-line option. Fluoxetine has displayed moderate effectiveness for repetitive behaviors in both children and adults with ASD. Other SSRIs, including fluvoxamine and citalopram, have shown mixed results, with some studies indicating minimal effects or poor tolerance. Sertraline and escitalopram have shown promise but lack specific measures of obsessive-compulsive symptoms in current research. Antiepileptic drugs such as valproate have shown some evidence of reducing repetitive behaviors in small studies. For treating schizophrenia in patients with ASD, antipsychotics are used to manage psychotic symptoms. Lithium, primarily a mood stabilizer, has demonstrated promise in patients with ASD, with one study indicating a 73.7% improvement rate in maladaptive behaviors among those receiving lithium treatment, particularly in individuals with ADHD characteristics. Additionally, valproate, functioning as both an anticonvulsant and mood stabilizer, has been shown to effectively reduce irritability in ASD without necessitating a specific bipolar disorder diagnosis [57,58,59,60,61,62,63,64,65,66,67,68,69,70,71,72,73,74,75,76,77,78,79,80,81,82,83,84,85,86,87,88,89,90,91,92,93,94,95,96,97,98,99,100,101,102,103,104,105,106,107,108,109,110,111,112,113,114,115,116,117,118,119,120,121,122,123,124,125,126,127,128,129,130,131,132,133,134,135,136,137,138,139,140,141,142,143,144].

## 2. Epilepsy

### 2.1. General Features

Seizures represent a complex phenomenon characterized by abnormal and unregulated electrical discharges occurring in the cortical gray matter of the brain. These episodes can lead to transient disruptions in normal brain function, manifesting as altered awareness, unusual sensations, focal involuntary movements, or, in severe cases, convulsions, which are marked by widespread and violent contractions of voluntary muscles. The diagnosis of seizures is primarily clinical, often supplemented by neuroimaging, laboratory tests, and electroencephalography (EEG), particularly in cases of new-onset seizures. For individuals with previously diagnosed seizure disorders, monitoring the levels of antiseizure medications (anticonvulsants) is essential. Management strategies for seizures focus on identifying and addressing underlying causes whenever possible. Treatment options typically include the use of antiseizure medications, and in cases where pharmacological interventions are ineffective, surgical options may be considered. Overall, effective management of seizures requires a comprehensive approach tailored to the individual patient, combining clinical assessment with appropriate diagnostic tools and therapeutic interventions [145,146].

Epilepsy, also referred to as epileptic seizure disorder, is a chronic neurological condition characterized by the occurrence of recurrent seizures that are unprovoked, defined as occurring at least two times and separated by a minimum of 24 h. It is important to note that a single seizure does not qualify for a diagnosis of epilepsy. While many cases of epilepsy are classified as idiopathic, meaning the exact cause remains unknown, there are instances where identifiable brain disorders, such as malformations, strokes, or tumors, lead to symptomatic epilepsy. Symptomatic epilepsy refers to seizure activity that can be directly attributed to a known underlying condition, with symptomatic epileptic seizures being more prevalent among neonates and older adults. In addition to symptomatic epilepsy, there exists a category known as cryptogenic epilepsy. This classification is used when a specific cause for the seizures is suspected but remains undetermined. It is crucial to differentiate between epileptic seizures and nonepileptic seizures, the latter of which are provoked by temporary conditions or stressors. These can include metabolic imbalances, central nervous system infections, cardiovascular issues, medication toxicity or withdrawal, and psychogenic disorders. In pediatric populations, fever can lead to febrile seizures, which, while seizure-like, are not classified as epilepsy. Moreover, psychogenic nonepileptic seizures, often referred to as pseudoseizures, are episodes that mimic seizures but occur in individuals with underlying psychiatric conditions. These episodes do not involve abnormal electrical discharges in the brain, distinguishing them from true epileptic seizures [145].

### 2.2. Etiology

Seizures and epilepsy arise from an imbalance between excitation and inhibition within specific regions of the central nervous system (CNS). Given the multitude of mechanisms that regulate neuronal electrical activity, various factors can disrupt this balance, leading to numerous causes of both seizures and epilepsy. The International League Against Epilepsy (ILAE) Task Force has delineated six etiologic categories for epilepsy: genetic, structural, metabolic, infectious, immune, and unknown. These categories are non-hierarchical, and a patient’s epilepsy may span multiple etiological classifications.

- *Structural etiology*:

Structural etiologies refer to identifiable abnormalities on neuroimaging that are reasonably inferred to contribute to a patient’s seizures, supported by concordant electroclinical assessments and clinical findings. These structural causes can be categorized as either acquired or genetic. Acquired structural etiologies encompass conditions such as hypoxic-ischemic encephalopathy, stroke, trauma, and infections. Common epileptogenic pathologic processes include also mesial temporal sclerosis, malformations of cortical development, focal encephalomalacia, primary brain tumors, vascular malformations, and neurocysticercosis. In contrast, genetic origins involve a wide array of disorders, including those from single-nucleotide mutations such as missense, frameshift, and nonsense mutations, copy number variations by de novo or inherited DNA deletion or duplication, and chromosomal copy number abnormalities [145,147].

- *Genetic etiology*:

Epilepsy of genetic origin is identified when a specific disease-causing variant in a gene or a copy number variant is known or presumed, with seizures being a common phenotype. Wang and his team identified 977 genes associated with epilepsy, categorizing them into four distinct groups based on their relationship to epilepsy phenotypes. These categories include epilepsy genes (which cause epilepsies or syndromes where epilepsy is the core symptom), neurodevelopment-associated epilepsy genes (linked to both brain developmental malformations and seizures), epilepsy-related genes (associated with systemic abnormalities alongside seizures), and genes putatively associated with epilepsy (which require further validation) [145,148,149].

Epilepsy can be classified into three categories: genetic generalized epilepsy (GGE), focal epilepsy (FE), and epileptic encephalopathy, each encompassing specific syndromes characterized by variations in seizure types, EEG patterns, age of onset, and disease progression [145,150].

GGE syndromes typically emerge during childhood or adolescence and are characterized by generalized seizures that engage both hemispheres of the brain. They include juvenile myoclonic epilepsy and childhood absence epilepsy, associated with large recurrent deletions at chromosomes 15q13.3, 16p13.11, and 15q11.2. These deletions have also been linked to conditions such as autism and schizophrenia [151,152]. Focal epilepsies, on the other hand, originate in one hemisphere of the brain and include familial mesial temporal lobe epilepsy (FMTLE), autosomal dominant lateral temporal epilepsy (ADLTE), and autosomal dominant nocturnal frontal lobe epilepsy (ADNFLE). FMTLE is a benign syndrome characterized by significant psychic and autonomic seizures, with no correlation to hippocampal sclerosis (HS) or febrile seizures (FS). Genetic studies have identified markers on chromosome 4q13.2–q21.3 [153]. ADLTE is characterized by heritable temporal lobe epilepsy with auditory ictal manifestations; mutations in the leucine-rich glioma-inactivated 1 (LGI1) gene at the 10q24 locus were identified through linkage analysis [154]. ADNFLE, which has childhood onset, is characterized by brief nocturnal motor seizures originating from the frontal lobe and was the first inherited epilepsy for which a specific mutation was identified. The gene implicated, CHRNA4, is located on chromosome 20q13 and encodes the α-4 subunit of the neuronal nicotinic acetylcholine receptor (nAChR). In patients with ADNFLE, a missense mutation results in the substitution of serine with phenylalanine at position 247, a highly conserved amino acid residue in the second transmembrane domain [155]. Epileptic encephalopathies represent a severe, early onset category of conditions characterized by refractory seizures, developmental delays, or regressions linked to ongoing epileptic activity, and generally poor prognoses. This category includes disorders attributed to mutations in genes encoding ion channels, such as KCNQ2 in benign familial neonatal seizures, SCN2A in benign familial infantile epilepsy, and SCN1A in Dravet syndrome. The KCNQ2 gene encodes voltage-gated potassium channels responsible for producing the M current, which normally induces a hyperpolarizing shift in membrane voltage. Loss of KCNQ2 function can lead to increased neuronal hyperexcitability, resulting in spontaneous seizure activities. The SCN1A gene encodes NaV1.1, one of nine α subtypes of voltage-gated sodium channels primarily expressed in GABAergic neurons. It is a critical causative gene in epilepsy. Dysfunction of Nav1.1 channels results in diminished excitability of GABAergic neurons, contributing to brain hyperexcitability in individuals with Dravet syndrome [145,156,157,158,159,160].

- *Infectious Etiology*:

Infections of the central nervous system (CNS) pose a significant risk factor for the development of epilepsy. They represent the most prevalent identifiable cause of this condition. The risk of unprovoked seizures among survivors of CNS infections ranges from 6.8% to 8.3%, with even higher rates observed in low- and middle-income countries (LMICs) [145,161]. An infectious etiology pertains specifically to patients with epilepsy that arises as a consequence of prior CNS infections rather than those experiencing seizures due to an acute CNS infection. In this context, seizures are induced by alterations in the brain caused by neurotropic infectious agents or post-infectious reaction (acute disseminated encephalomyelitis, ADEM), such as cysticercus, human immunodeficiency virus (HIV), cytomegalovirus, measles, chickenpox, Toxoplasma gondii, Mycobacterium tuberculosis, and Plasmodium falciparum. Each infectious agent leads to distinct forms of cerebral damage, such as cortical necrosis associated with viruses, infarction resulting from bacterial meningitis, hypoxic–ischemic injury due to cerebral malaria, and gliosis surrounding calcified larvae in neurocysticercosis. Additionally, these infections provoke an immune and inflammatory response in the affected brain tissue [145]. The sustained activation of pro-inflammatory signals, whether from chronic inflammation or from seizures themselves, can result in damage to the blood–brain barrier (BBB), neuronal death, and persistent neuronal hyperexcitability. Furthermore, immune responses to systemic (non-CNS) infections may induce alterations in BBB integrity through pro-inflammatory cytokines, leading to subsequent neuronal hyperexcitability [161].

- *Metabolic etiology*:

Metabolic epilepsy is characterized by seizures that directly arise from known or presumed metabolic disturbances. In contrast, individuals experiencing transient metabolic disturbances that lead to acute symptomatic seizures do not qualify, as these seizures are provoked and thus not classified as epileptic. Various metabolic disorders, often stemming from genetic abnormalities, can result in cellular degeneration, dysmyelination, or disorders of neuronal migration, thereby indirectly promoting epileptogenesis by adversely affecting cellular or organ function. These disorders are categorized into small- or large-molecule disorders. Small-molecule disorders encompass issues related to amino acids, organic acids, fatty acids, neurotransmitters and their metabolites, urea cycle constituents, and various vitamins and cofactors. They are categorized as aminoacido-pathies, organic acidemias, demyelinating conditions, defective GABA metabolism, and mitochondrial disorders. Large-molecule disorders, on the other hand, include lysosomal storage diseases, peroxisomal disorders, glycosylation disorders, and leukodystrophies. Although most metabolic epilepsies have a genetic basis, some can be acquired, such as pyridoxine-dependent seizures and cerebral folate deficiency [145,149,162].

- *Immune etiology*:

An immune etiology should be considered in patients with epilepsy of unknown origin, especially when they test positive for neural-specific antibodies and exhibit signs of autoimmune-mediated CNS inflammation. Autoimmune epilepsies account for approximately 5–7% of all epilepsy cases [163]. Recognizing this etiology is crucial for treatment, as seizures triggered by autoimmune encephalitis require immunotherapies. Autoimmune epilepsy has been associated with various neuronal cell surface antigens, including LGI1, N-methyl-D-aspartate receptor (NMDA-R), α-amino-3-hydroxy-5-methyl-4-isoxazolepropionic acid (AMPA), GABA-B, and metabotropic glutamate receptor 5 (mGluR5), as well as intracellular antigens like glutamic acid decarboxylase 65 (GAD65), antineuronal nuclear antibody type 1 (ANNA-1), and small cell lung carcinoma (Ma). Autoantibodies targeting plasma membrane epitopes appear to disrupt ion channel functions; for instance, IgG anti-LGI1 interferes with the LGI1–ADAM22 interaction, leading to diminished synaptic AMPA receptor function and reduced calcium influx. Similarly, IgG anti-NMDA-R binds to a specific region of the GluN1 subunit of NMDA-R, disrupting the interaction between NMDA-R and ephrin type B2 receptor, which is also associated with decreased synaptic NMDA-R-mediated currents. In contrast, autoantibodies against intracellular targets are thought to mediate their pathogenic effects through cytotoxic T cells, resulting in neuronophagia, granzyme B neurotoxicity, neuronal loss, and gliosis, all of which can promote epileptogenesis in affected individuals. Immune responses play a significant role not only in seizure induction but also in the development of epilepsy. Both innate (inflammation) and adaptive immune responses are activated within the epileptic brain by resident immune cells and their secreted mediators, as well as by leukocytes that migrate from the periphery. The neuroinflammatory processes can originate either peripherally or centrally. Peripheral inflammation can enhance epileptic discharges by altering ion and glutamate homeostasis and facilitating the migration of pro-inflammatory molecules from peripheral inflammatory sites to the blood–brain barrier (BBB). The initial pathological triggers may include febrile seizures, trauma, stroke, or infections, any of which can initiate an inflammatory cascade. This cascade involves the activation of the IL-1 receptor/toll-like receptor (IL-1R/TLR) signaling pathways via ligation of pathogen-associated molecular patterns (PAMPs) or damage-associated molecular patterns (DAMPs). It also activates the cyclooxygenase-2 (COX-2) pathway and initiates the transforming growth factor-β/small mothers against decapentaplegic (TGF-β/Smad) signaling cascade. The activation of glial cells, neurons, and endothelial cells that form the BBB likely leads to the release of pro-inflammatory cytokines, such as IL-1β and TNF-α, along with danger signals like high-mobility group box-1 (HMGB1). These inflammatory mediators contribute to BBB permeability, enhance its leakage, and upregulate leukocyte adhesion molecules on the endothelium, which attracts lymphocytes from the adaptive immune system, resulting in their infiltration into the CNS. Lastly, there exists a sixth category of etiology, termed unknown, which is designated for patients whose etiology remains undetermined [163,164,165,166,167].

### 2.3. Classification of Seizure Disorders

The International League Against Epilepsy (ILAE) provides a comprehensive classification system for seizure disorders that is essential for clinicians in diagnosing and managing epilepsy. This classification system has evolved over the years, with the last update being in 2017. The ILAE classification system categorizes seizures based on clinical and electroencephalographic (EEG) features, as well as the underlying etiology. The classification has three primary components: seizure types, epilepsy types, and epilepsy syndromes.

- Seizure type: Seizures are classified into the following:
  ○ Focal seizures: the seizure originate in a specific area of the brain. These can be further divided into the following:
    ▪ Focal onset aware seizures
    ▪ Focal onset impaired awareness seizures
    ▪ Focal to bilateral tonic-clonic seizures
  ○ Generalized seizures: the seizures involve both hemispheres of the brain from the onset and include the following:
    ▪ Tonic-clonic seizures
    ▪ Absence seizures
    ▪ Myoclonic seizures
    ▪ Atonic seizures.
  ○ Unknown seizures
- Epilepsy Types
- Epilepsy syndromes: classified based on the seizure types, their clinical features, age of onset, and associated conditions.

#### 2.3.1. Focal-Onset Seizures

Focal-onset seizures originate from one hemisphere and may originate in subcortical structures. They may be discretely localized or more widely distributed.

Focal-onset seizures may be classified as the following:

- Focal aware seizures (formerly simple partial seizures)
- Focal impaired-awareness seizures (formerly complex partial seizures)

If awareness is impaired during any part of the seizure, the seizure is classified as a focal impaired-awareness seizure.

Focal-onset motor seizures may be further classified into the following:

- Automatisms (coordinated, purposeless, repetitive motor activity)
- Atonic (focal loss of muscle tone)
- Clonic (focal rhythmic jerking)
- Epileptic spasms (focal flexion or extension of arms and flexion of trunk)
- Hyperkinetic (causing pedaling or thrashing)
- Myoclonic (irregular, brief focal jerking)
- Tonic (sustained focal stiffening of one limb or one side of the body)

Focal-onset nonmotor seizures may be further classified into the following:

- Autonomic dysfunction (autonomic effects such as gastrointestinal (GI) sensations, a sense of heat or cold, flushing, sexual arousal, piloerection, and palpitations)
- Behavior arrest (cessation of movement and unresponsiveness as the main feature of the entire seizure)
- Cognitive dysfunction (impairment of language or other cognitive domains or positive features such as déjà vu, hallucinations, illusions, or perceptual distortions)
- Emotional dysfunction (manifesting with emotional changes, such as anxiety, fear, joy, other emotions, or affective signs without subjective emotions)
- Sensory dysfunction (causing somatosensory, olfactory, visual, auditory, gustatory, or vestibular sensations or a sense of heat or cold)

Focal-onset seizures may evolve into a generalized-onset tonic-clonic seizure (called a focal-to-bilateral tonic-clonic seizure; formerly, secondary generalization), which causes loss of consciousness. Focal-to-bilateral tonic-clonic seizures occur when a focal-onset seizure spreads and activates the entire cerebrum bilaterally. Activation may occur so rapidly that the initial focal-onset seizure is not clinically apparent or is very brief [146].

#### 2.3.2. Generalized-Onset Seizures

In generalized-onset seizures, seizures originate from both hemispheres. Awareness is usually impaired, and consciousness is usually lost. They are classified as motor or nonmotor (absence) seizures. Motor activity is usually bilateral from the onset.

- Generalized-onset motor seizures may be further classified into the following:
  ○ Tonic-clonic seizures (formerly grand mal seizures)
  ○ Clonic seizures (sustained rhythmic jerking)
  ○ Tonic seizures (generalized stiffening involving all limbs and without rhythmic jerking)
  ○ Atonic seizures (loss of muscle tone)
  ○ Myoclonic seizures (rhythmic jerking not preceded by stiffening)
  ○ Myoclonic-tonic-clonic seizures (myoclonic jerking followed by tonic and clonic movements)
  ○ Myoclonic-atonic seizures (myoclonic jerking followed by atonia)
  ○ Epileptic spasms (formerly, infantile spasms)
- Generalized-onset nonmotor seizures may be further classified into the following:
  ○ Typical absence seizures
  ○ Atypical absence seizures (with less abrupt onset or termination or with abnormal changes in tone)
  ○ Myoclonic seizures (myoclonic jerking preceded by brief impairment of consciousness)
  ○ Eyelid myoclonia [146].

#### 2.3.3. Unknown-Onset Seizures

Seizures are usually classified as unknown-onset seizures when information about onset is lacking.

Seizures of unknown onset can be motor or nonmotor.

Unknown-onset motor seizures may be further classified as

- Tonic-clonic
- Epileptic spasms

Unknown-onset nonmotor seizures may be further classified as

- Behavior arrest [146].

### 2.4. Clinical Features

Seizures are neurological events that can manifest in various forms, often preceded by an aura, which describes the sensations or feelings experienced by patients before a seizure begins. Auras may include motor activity; sensory, autonomic, or psychic sensations, such as paresthesia; rising epigastric sensations; abnormal smells; feelings of fear; and phenomena like déjà vu or jamais vu. The latter refers to a familiar experience feeling strangely unfamiliar. Typically, auras are associated with focal aware seizures.

Most seizures resolve spontaneously within 1 to 2 min. After a seizure, particularly generalized-onset seizures, patients often enter a postictal state characterized by deep sleep, confusion, headache, and muscle soreness, lasting from minutes to hours. This state may also involve Todd paralysis, a temporary neurologic deficit, usually resulting in weakness of the limb opposite to the seizure focus.

Patients generally appear neurologically normal between seizures, although high doses of sedating antiseizure medications can impair alertness. Any progressive cognitive decline is usually linked to the underlying neurologic disorder rather than the seizures themselves. In rare cases, seizures can become unremitting, leading to status epilepticus [146].

Focal-onset seizures are categorized in focal aware seizures, formerly known as simple partial seizures, if during the seizure awareness is maintained and focal impaired-awareness seizures, formerly known as complex partial seizures, if awareness is impaired, though not completely lost.

Focal aware seizures can produce motor, sensory, or psychomotor symptoms that reflect the affected brain region. For example, Jacksonian seizures exhibit motor symptoms starting in one hand and progressing up the arm (Jacksonian march). Other types of focal-onset seizures may begin in the face before spreading to limbs.

Epilepsia partialis continua is a rare condition characterized by continuous focal aware motor seizures, typically involving one arm, hand, or side of the face. In adults, it often results from a structural lesion, while in children, it may stem from a focal cerebral cortical inflammatory process such as Rasmussen encephalitis. Focal-onset seizures in patients can manifest transient, unexplained psychiatric-like symptoms such as hallucinations, behavioral automatisms, or speech arrest.

During focal impaired-awareness seizures, patients may exhibit oral or limb automatisms, stare blankly, resist assistance, and show posturing of the limb opposite to the seizure focus. Afterward, confusion and disorientation can persist for several minutes, with postictal amnesia being common.

Generalized-onset seizures typically involve impaired awareness from the outset and can be categorized as motor or nonmotor (absence) seizures. Absence seizures can be classified into typical absence seizures, if the seizure is characterized by several seconds to 30 s of loss of consciousness and eyelid fluttering, or atypical absence seizures, if the seizures last longer, with more pronounced jerking or automatic movements and less complete loss of awareness. Typical absence seizures predominantly affect children and often do not continue into adulthood. They may be triggered by hyperventilation and usually do not present with postictal symptoms. Atypical absence seizures are frequently seen in Lennox–Gastaut syndrome, a severe epilepsy form typically beginning before age 4, marked by various seizure types and associated with cognitive impairment and behavioral issues. Myoclonic absence seizures involve rhythmic jerking of the arms and shoulders, with the seizure lasting about 10–60 s, while atonic seizures result in a brief and complete loss of muscle tone, posing a risk of head injury, especially in children.

Eyelid myoclonia consists of myoclonic jerks of the eyelids and upward deviation of the eyes, usually triggered by closing eyes or exposure to light. It can manifest during both motor and non-motor seizures.

Tonic seizures primarily occur during sleep and are most commonly seen in children often associated with Lennox–Gastaut syndrome. These seizures involve a sustained contraction of the axial muscles, which can start either abruptly or gradually and may then extend to the proximal muscles of the limbs. During a tonic seizure, the neck typically becomes stiff. The duration of tonic seizures is generally between 10 and 15 s, although longer episodes may be followed by a few rapid clonic jerks as the tonic phase concludes.

Clonic seizures are characterized by sustained, rhythmic jerking movements that affect both sides of the body, including the limbs as well as the head, neck, face, and trunk. Clonic seizures are most frequently observed in infants and should be differentiated from jitteriness or shuddering attacks. Clonic seizures are significantly less common than tonic-clonic seizures.

Tonic-clonic seizures can be classified as generalized-onset tonic-clonic seizures, formerly known as primarily generalized seizure, which typically last 1–2 min and start with an outcry leading to loss of consciousness and falling with tonic contraction followed by clonic (rapidly alternating contraction and relaxation) motion of the muscles of the extremities, trunk, and head and may involve tongue biting and incontinence, both urinary and fecal, as well as frothing at the mouth, and focal-to-bilateral tonic-clonic seizures, formerly known as secondarily generalized seizures, which begin with a focal aware or impaired-awareness seizure before progressing to a generalized tonic-clonic seizure.

Myoclonic seizures consist of brief jerks of the limbs or the trunk, which may evolve into generalized tonic-clonic seizures. Myoclonic-atonic seizures involve brief jerks followed by limpness (drop attacks), commonly observed in young children.

Epileptic spasms, previously known as infantile spasms, manifest as sudden flexion and adduction of the arms and trunk flexion, occurring in infants and often associated with developmental defects.

Juvenile myoclonic epilepsy typically arises during adolescence. It is characterized by myoclonic and tonic-clonic seizures, often triggered by sleep deprivation or alcohol use.

Febrile seizures occur in children with fever, are typically brief, and are classified as benign or complicated based on duration and characteristics. Complicated febrile seizures carry a higher risk of subsequent seizure disorders.

Dravet syndrome presents in early childhood with a mix of focal and generalized seizures, often related to mutations in the SCN1A gene.

Status epilepticus is defined as continuous seizure activity and can be classified into convulsive and nonconvulsive forms. Convulsive status epilepticus involves prolonged tonic-clonic activity or multiple seizures without full recovery of consciousness. Untreated generalized seizures lasting over 60 min can lead to permanent brain damage or death.

Nonconvulsive status epilepticus includes focal-onset status epilepticus and absence status epilepticus, with prolonged episodes of altered mental status, often requiring EEG for diagnosis [146,149,168,169,170].

#### Sudden Unexpected Death in Epilepsy (SUDEP)

SUDEP is a rare but serious complication of seizures, typically occurring during sleep. The risk is heightened in patients with frequent seizures, particularly generalized tonic-clonic seizures. While no definitive measures prevent SUDEP, optimal seizure control is the recommended strategy [146,171].

### 2.5. Diagnostic Procedures

Clinical evaluation of seizures is critical for determining whether an event is a seizure or due to other causes of altered consciousness, such as pseudoseizures or syncope. Patients typically undergo neuroimaging (CT or MRI), laboratory testing, and EEG in an emergency department setting. For known seizure disorders, patients are often evaluated in outpatient settings with a focus on monitoring antiseizure medication levels to avoid toxicity. A comprehensive history is vital for understanding seizure characteristics and potential risk factors. In anamnesis, clinicians must collect information both from the patient and eventually from the witness about seizure description, such as duration, frequency, evolution, presence of aura, postictal state, risk factors, prior head trauma, CNS infections, neurological disorders, substance use/withdrawal, nonadherence to medications, family history of seizures, trigger events, such as repetitive sounds, flashing lights, video games, and sleep deprivation, etc. In the physical examination, the clinician should look for evidence such as tongue biting, incontinence, and prolonged confusion postictally, longer duration, absence of postictal confusion, atypical progression of muscular activity, and resistance to eye opening (pseudoseizure indicator). Routine testing is essential, although normal results do not rule out the presence of seizures. Testing should be guided by clinical findings. Other tests required by clinicians for differential diagnosis include lumbar puncture to exclude meningitis or CNS infection, drug screens to exclude illicit use of drug, and researching for anti-NMDA receptor antibodies, especially in young women. Other required procedures include CT, the first-line imaging modality to rule out hemorrhage or mass lesions; MRI if CT results are negative, providing better resolution for soft tissue abnormalities; and EEG, crucial for diagnosing epileptic seizures, particularly focal impaired-awareness seizures. For surgical candidates, a functional MRI, magnetoencephalography, and SPECT are usually required, which can help localize seizure foci for potential surgical intervention and neuropsychological testing, which is crucial to evaluate cognitive deficits and predict rehabilitation potential post-surgery [146,172,173,174,175].

### 2.6. Treatment

Refs. [176,177,178,179,180,181,182,183,184,185,186,187,188,189,190,191,192,193,194,195,196,197,198,199,200,201,202,203,204,205,206,207,208,209,210,211,212,213,214] Anti-epileptic drugs (AEDs) encompass a wide range of medications [Table 3].

Certain AEDs target sodium channels by blocking their repeated activation, such as phenytoin and carbamazepine, or by enhancing their slow inactivation, such as lacosamide. Other medications focus on calcium channels, blocking T-type calcium channels, such as ethosuximide and valproic acid, or N- and L-type calcium channels, such as zonisamide. Lamotrigine blocks sodium channels, inhibits N- and L-type calcium channels, and modulates H-currents. Topiramate blocks sodium channels and AMPA receptors and inhibits carbonic anhydrase. Phenobarbital and benzodiazepines enhance GABA-A receptor function. Felbamate blocks NMDA receptors, while ezogabine opens potassium channels [176,177].

- Carbamazepine (C_15_H_12_N_2_O, CBZ) is a dibenzazepine derivative used for both focal and generalized seizures for the treatment of bipolar disorder and trigeminal neuralgia. It works by stabilizing the inactivated state of voltage-gated sodium channels in neuronal cell membranes, and by inhibiting these channels, it reduces excessive neuronal firing that is characteristic of epilepsy and can also stabilize mood in bipolar disorder. Additionally, it may influence neurotransmitter systems and has been shown to have some effect on gamma-aminobutyric acid (GABA) receptors. Carbamazepine is well absorbed from the gastrointestinal tract, is extensively bound to plasma proteins and has a large volume of distribution, is primarily metabolized in the liver by cytochrome P450 enzymes (mainly CYP3A4), has an active metabolite, carbamazepine-10,11-epoxide, and has an elimination half-life of up to 35 h initially that decreases with chronic use due to autoinduction of its own metabolism. Common side effects include gastrointestinal symptoms, hyponatremia, rash, itching, drowsiness, dizziness, blurred and double vision, headache, and ataxia. More serious but rare side effects can include Stevens–Johnson syndrome, toxic epidermal necrolysis, leukopenia, aplastic anemia (pancytopenia), agranulocytosis, and hepatic dysfunction. Patients in treatment must be monitored for complete blood counts, liver function tests, serum sodium levels, and therapeutic drug levels [176,178,179,180,181,182,183].
- Oxcarbazepine (C_15_H_14_N_2_O_2_) is a keto-derivative of carbamazepine and has a comparable mechanism of action, working by stabilizing hyperexcitable neuronal membranes and inhibiting repetitive neuronal firing. It is used for the treatment of focal and secondarily generalized tonic-clonic seizures in adults and children aged 2 years and older and may also be used off-label for the treatment of neuropathic pain and as a mood stabilizer in bipolar disorder. Common side effects include dizziness, headache, ataxia, nausea, rash, double vision, and hyponatremia, which may occur due to the syndrome of inappropriate secretion of antidiuretic hormone (SIADH). Regular monitoring of sodium levels is recommended due to the risk of hyponatremia. Caution should be taken in patients with a history of allergic reactions to carbamazepine, as cross-reactivity may occur [176,180,184,185,186,187].
- Phenytoin (C_15_H_12_N_2_O_2_) is a hydantoin derivative, one of the oldest anti-seizure medications. It is widely used for focal and generalized seizures and is also indicated for status epilepticus. It may serve as a second-line agent for mixed seizure types such as tonic-clonic and myoclonic. It is rapidly absorbed from the gastrointestinal tract, has highly protein-bound (approximately 90–95%) and a large volume of distribution, is metabolized in the liver via cytochrome P450 enzymes, particularly CYP2C9 and CYP2C19, and has a half-life typically ranging from 7 to 42 h, influenced by dosage and individual patient factors. Phenytoin primarily blocks voltage-gated sodium channels, but it may also exert effects through decreased synaptic transmission and alterations in ionic gradients and induces the CYP enzyme system, which can diminish the effectiveness of various hormonal oral contraceptives. Significant adverse effects include gingival hyperplasia, hirsutism, folic acid depletion, rash, and nausea, as well as potential declines in bone density. Long-term use may lead to dizziness, drowsiness, ataxia, double vision, osteoporosis, hepatic dysfunction, and neuropathy. It is contraindicated for patients with hypersensitivity to hydantoins and phenytoin. Caution must be also taken in patients with liver disease. Regular monitoring of serum phenytoin levels is essential to ensure therapeutic efficacy and minimize toxicity [180,188,189,190,191].
- Lacosamide (C_13_H_18_N_2_O_3_) stabilizes hyperexcitable membranes and inhibits repetitive neural firing through slow inactivation of sodium channels. It may also bind to collapsing response mediator protein 2 (CRMP2), which is linked to neuronal growth and differentiation, involved in the epileptogenesis process. It is well absorbed after oral administration, with peak plasma concentrations occurring approximately 1 to 4 h after a dose, has a volume of distribution of approximately 0.6 L/kg, is approximately 15% bound to plasma proteins, undergoes hepatic metabolism primarily through CYP2C19, with minimal contribution from CYP3A4 and CYP2C9, and has an elimination half-life of about 12 to 15 h. It is approved for use in patients aged four and older, as monotherapy or adjunct therapy for focal-onset seizures. It is generally well tolerated, with dizziness, headache, nausea, fatigue, diplopia, and ataxia as the most common side effects. It can cause dose-dependent PR interval prolongation on electrocardiograms, necessitating EKG (electrocardiogram) monitoring at treatment initiation, and is contraindicated in patients with a history of cardiac conduction abnormalities. It is also contraindicated in patients with known hypersensitivity to lacosamide or any of its components [192,193,194,195,196].
- Phenobarbital (C_12_H_12_N_2_O_3_) is a barbiturate derivative, utilized for both generalized and focal seizures, to manage insomnia, and for preoperative sedation. It works by enhancing the activity of gamma-aminobutyric acid (GABA) at the GABA-A receptor, prolonging its open state to increase chloride influx and hyperpolarize cells and leading to increased inhibitory neurotransmission in the central nervous system (CNS). Its use is very limited in modern times due to its sedative effects and the risk of dependence. Phenobarbital is well-absorbed from the gastrointestinal tract after oral administration. It can also be given intravenously and intramuscularly. It is widely distributed throughout the body, including the brain, has a high volume of distribution, is primarily metabolized in the liver via cytochrome P450 enzymes, and has an elimination half-life that ranges from 80 to 120 h, which can be prolonged in cases of liver impairment or in neonates. Common collateral effects are drowsiness, dizziness, and cognitive impairment, particularly at higher doses. Severe side effects are risk of respiratory depression, especially when combined with other CNS depressants, and dependence and withdrawal symptoms with long-term use [197,198,199].
- Vigabatrin (C_8_H_13_N_3_O_3_S, 4-amino-hex-5-enoic acid) is a derivative of gamma-aminobutyric acid (GABA), an irreversible inhibitor of the enzyme GABA transaminase, an enzyme responsible for GABA metabolism, raising GABA levels in the central nervous system. It serves as an adjunct treatment for refractory focal seizures and can also be used as monotherapy for the same indication. Additionally, it is also used in pediatric patients with infantile spasms, especially in those with tuberous sclerosis complex. It is rapidly absorbed after oral administration, with peak plasma concentrations occurring within 1 to 2 h, is widely distributed in body tissues, with a volume of distribution of approximately 0.6 L/kg, undergoes minimal hepatic metabolism, and has an elimination half-life of approximately 5 to 10 h. Common side effects are peripheral vision loss, fatigue, dizziness, headache, weight gain, behavioral changes such as irritability and agitation, nausea, and MRI abnormalities without clear neurological deficits [200,201,202,203,204].
- Topiramate (C_12_H_21_N_3_O_8_S) blocks voltage-gated sodium channels, enhances GABA transmission, antagonizes NMDA receptors (AMPA/kainate subtype of glutamate receptors), and has a mild inhibiting action on carbonic anhydrase. It is approved for the treatment of focal-onset and primary generalized tonic-clonic seizures (in both children and adults ages 10 and up) and as adjunctive therapy for focal seizures or Lennox–Gastaut syndrome (ages 2 and up). Patients may experience weight loss, cognitive impairment, expressive language difficulties, fatigue, taste changes, nausea depression, headache, and paresthesia. There is also a risk of metabolic acidosis with tachypnea and calcium phosphate kidney stones and of suicidal thoughts or behavior [180,205].
- Valproate (C_8_H_15_NaO_2_ valproic acid, VPA) acts by blocking sodium channels, increasing GABA levels, and mildly inhibiting T-type calcium currents. It is used for both focal and generalized seizures. It is rapidly absorbed from the gastrointestinal tract, with peak plasma concentrations typically reached within 1–4 h after oral administration, is widely distributed in body tissues, with a volume of distribution ranging from 0.1 to 0.5 L/kg, and is highly protein-bound (approximately 90–95%). It is metabolized in the liver through glucuronidation and beta-oxidation and has an elimination half-life of 9–16 h. Common side effects include nausea, tremor, easy bruising, weight gain, insulin resistance, metabolic syndrome, and subclinical hypothyroidism. Rare but serious side effects can include acute hepatocellular injury with jaundice and acute pancreatitis. VPA is contraindicated during pregnancy due to its teratogenic effects [206,207,208,209,210].
- Levetiracetam (C_8_H_14_N_2_O_2_) is a pyrrolidine derivative whose exact mechanism of action is not fully understood, but it is believed to involve the inhibition of synaptic vesicle protein 2A (SV2A). It is used as adjunctive therapy for focal-onset seizures in both children and adults, myoclonic seizures in juvenile myoclonic epilepsy (ages 12 and up), and primary generalized tonic-clonic seizures (ages 6 and up) in patients with idiopathic generalized epilepsy. Levetiracetam is rapidly absorbed following oral administration, with peak plasma concentrations occurring within 1 to 2 h, has a bioavailability of approximately 100% and a volume of distribution of about 0.5 to 0.7 L/kg, and is approximately 10% protein-bound. Levetiracetam is minimally metabolized by the liver and has an elimination half-life of approximately 7 h. Common side effects include behavioral changes, fatigue, headache drowsiness, dizziness, upper respiratory infections, mood disorders, and neurocognitive issues. Side effects associated with AEDs can range from 7% to 31%, though most are mild and reversible. Allergic reactions are a potential concern with all medications and warrant monitoring upon initiation. With chronic use, levetiracetam can lead to osteoporosis; therefore, patients are advised to supplement with calcium and vitamin D and maintain regular exercise. Serious side effects can include Stevens–Johnson syndrome, agranulocytosis, aplastic anemia, hepatic failure, pancytopenia, multiorgan hypersensitivity, psychosis, and lupus syndrome [180,207,208,209,210,211,212,213,214].

Once an AED treatment has been initiated, it is recommended to monitor serum medication levels to establish a therapeutic baseline and check for toxicity. If the patient remains stable, these levels can be assessed annually, alongside complete blood counts, comprehensive metabolic panels, and liver function tests. Toxic symptoms from AEDs are often idiosyncratic and are more frequently associated with first-generation AEDs, such as carbamazepine, which can cause acute or chronic toxicity. Symptoms may include ataxia, dystonia, sinus tachycardia, hyperthermia, coma, arrhythmias, respiratory depression, and even death. Valproic acid can also lead to toxicity, presenting as metabolic and hematological disruptions, pancreatitis, CNS depression, optic nerve atrophy, respiratory issues, cardiopulmonary arrest, brain edema, and coma. Treatment for toxicity may range from supportive care to high-flow hemodialysis, plasmapheresis, or charcoal hemoperfusion. For children, it is generally recommended to consider discontinuing AEDs after two years of being seizure-free. In contrast, adults face a higher estimated risk of seizure recurrence (around 30%) immediately after stopping AEDs at the two-year mark. This risk appears to decrease over time for those who remain seizure-free, explaining why guidelines for adults suggest a more conservative approach of a seizure-free period of 2 to 5 years and recommend against driving for three months following AED cessation [176].

## 3. Epilepsy and Autism: An Overlapping Syndrome

### 3.1. Etiopathology and Pathophysiology of the Co-Occurrence of Epilepsy and ASD

Epilepsy is more common in autistic individuals than in the general population. The prevalence of epilepsy in autistic individuals in clinical sample-based studies was higher than that in population-based cross-sectional or cohort studies. The prevalence of epilepsy in autistic adults was higher than that in autistic children. A significantly increased prevalence of epilepsy was detected in the autistic adolescent group (11–17 years old), and a higher trend of prevalence of epilepsy was observed in the autistic pre-school group (≤6 years old) than that in the autistic school-aged group (7–10 years old). The prevalence of epilepsy increased with age, female rate, and low intellectual function rate of autistic individuals [215]. There is a suggested common biological mechanism linking childhood epilepsy and comorbid ASD, which may involve overlapping genetic subgroups contributing to similar phenotypic manifestations and biological functions. This association has been recognized since the late 1980s, particularly in children presenting with infantile spasms and associated ASD in the context of tuberous sclerosis complex (TSC). Refs. [216,217] Subsequent research has confirmed this connection by frequently identifying early developmental markers of ASD, such as deficits in social communication, in the first year of life in infants with TSC-related epilepsy [218,219,220]. This concept has been explored through extensive research, which over time has led to the term developmental and epileptic encephalopathy (DEE), the most severe group of epilepsies where early onset epilepsy with genetic etiology is associated with cognitive or behavioral disturbances such as ASD. More than 900 genes have been identified as monogenic causes of developmental and epileptic encephalopathies [221]. Molecular products involved in synaptic function and plasticity—such as genes encoding neuroligins and neurexins and neuronal activity-related genes like methyl CpG binding protein 2 (MECP2, linked to classic and atypical Rett syndrome (RTT), non-specific intellectual disability, cognitive regression and ASD, fatal neonatal encephalopathy, parkinsonism, pyramidal signs, and neuropsychiatric symptoms (such as bipolarism), and ubiquitin-protein ligase E3A (UBE3A, responsible for some cases of Angelman syndrome and Prader–Willi syndrome if lacking and autism if overexpressed), brain development genes like ARX (related to agenesis of the corpus callosum, ACC, learning disabilities, ASD, and epilepsy) and FOXP2 (linked to speech and language disorders, oral-motor dyspraxia, dysarthria, fine motor difficulties, and ASD), and those related to ion channel function (particularly sodium and calcium channels)—may underpin the common pathological processes observed in childhood epilepsy and comorbid ASD [216,222,223,224,225,226,227,228,229,230,231,232]. Structural or developmental lesions, genetic susceptibilities, and environmental factors may contribute to early onset epilepsy and ASD. There is a bidirectional relationship between epilepsy and ASD. Epilepsy may contribute to the development of ASD, or, alternatively, the underlying abnormal brain circuitry associated with ASD may predispose individuals to seizures. Aberrant protein translation may lead to brain overgrowth and altered connectivity, characteristics of these syndromes. Disruptions in the timing of synaptogenesis during early brain development may serve as mechanisms for the emergence of both epilepsy and ASD. An accelerated rate of cell and synaptic growth during the first three years of life, combined with heightened inhibitory currents, could increase the risk of developing ASD. Genetic mutations within the mammalian target of rapamycin (mTOR) and phosphoinositide 3-kinases (PI3K) pathways have been implicated in excessive synaptic and cellular growth. Mutations in the neurexin-neuroligin-SH3 and multiple ankyrin repeat domains 3 (NRXN–NLGN–SHANK) pathway are believed to disrupt synaptogenesis and contribute to an imbalance of inhibitory currents. Disruption of the balance between neuronal excitation and inhibition is recognized as a precursor to seizures and epilepsy. Increased excitation may arise from dysfunctions in ion channels responsible for depolarization such as in sodium or calcium channelopathies or enhancements in excitatory neurotransmission. Conversely, decreased inhibitory function, possibly due to potassium channelopathies or GABAergic dysfunctions in case of reduced synthesis, increased degradation, or impaired receptor activity, may also facilitate seizure onset. Atypical neuronal networks resulting from imbalances in excitation and inhibition, due to disrupted synaptic formation and development, could underlie core symptoms of ASD, such as impaired communication, aberrant socialization, and restricted behaviors or stereotypies [216]. Genetic mutations associated with childhood epilepsy and ASD frequently involve mechanisms that extend beyond ion channels and synaptic physiology, encompassing regulation of synaptic vesicle release, modulation of subcellular signaling pathways, and organization of neuronal network connections. Three main categories of risk genes may shed light on the epilepsy-ASD phenotype as a connectivity issue: (a) ion channels, (b) synaptic function and structure, and (c) transcriptional regulators. Numerous studies support the role of genes encoding ion channels (such as SCN1A, SCN2A, and SCN8A for sodium channels, KCNB1 for potassium channels, and CACNA1A for calcium channels) as leading risk factors for the epilepsy-ASD phenotype. Genes involved in synaptic function and structure, such as SHANK3, CDKL5, STXBP1, NRXN1, PCDH19, and CASK, are also significant. Furthermore, epigenetic modifications affecting DNA transcription are relevant factors in the occurrence of ASD, with genes like SYNGAP1, YWHAG, PACS2, ACTL6B, MEF2C, MECP2, and FOXG1 playing a role. Dysregulation of cellular growth is another crucial mechanism during early brain development that can lead to abnormal neuronal cytoarchitecture and, consequently, altered neuronal connectivity. For instance, the mTOR (mammalian target of rapamycin) pathway is involved in cell growth and proliferation, regulating neuronal morphology, GABAergic interneuron development, white matter connectivity, and the number and shape of synapses. Disruptions in this delicate pathway can lead to childhood epilepsy, intellectual disability, and ASD [216,233,234,235,236,237,238,239,240,241,242,243,244,245,246,247,248,249,250,251,252,253,254,255,256,257,258,259,260,261,262,263,264,265,266,267,268,269,270,271,272].

### 3.2. Treatment for Epilepsy in ASD Patients

Refs. [62,80,180,273,274,275,276,277,278,279,280,281,282,283,284,285,286,287,288,289,290,291,292,293,294,295,296,297,298,299,300] The pharmacological management of pediatric patients with ASD and comorbid epilepsy presents a multifaceted challenge. A tailored treatment plan is essential to effectively address the unique needs of each child. The co-occurrence of ASD and epilepsy is associated with a diverse array of symptoms that complicate pharmacological care. Experts often recommend initiating treatment by focusing on the management of ASD-related symptoms, utilizing medications designed to mitigate or alleviate these manifestations. Commonly prescribed medications include antipsychotics and antidepressants, such as risperidone, aripiprazole, or SSRIs, which modulating neurotransmitter systems such as dopamine and serotonin systems and stabilize neuronal excitability, reducing the likelihood of seizure activity. The selection of antiepileptic drugs (AEDs) is influenced by factors such as the type of seizure, the patient’s age, and any associated comorbidities, such as epileptic patients without ASD. In instances where individuals with ASD are not experiencing seizures, the focus of pharmacological interventions shifts to managing behavioral, emotional, or cognitive challenges such as anxiety, hyperactivity, aggression, and sleep disturbances. It is common for psychiatric medications to be used in conjunction with other drug classes, such as anxiolytics, antipsychotics, and antidepressants, to address both the psychiatric and epileptic symptoms, particularly in cases of severe epilepsy. A notable challenge in this therapeutic landscape is the potential for significant drug interactions among the various medications employed. Antipsychotic medications can exhibit important interactions with anticonvulsants and mood stabilizers. Carbamazepine, phenytoin, and phenobarbital, for example, are inducers of the cytochrome P450, so they can decrease the plasma concentrations of certain psychiatric medications such as antidepressants like tricyclics, SSRIs, and antipsychotics, potentially leading to reduced efficacy of medication, while valproate, on the other hand, is an inhibitor that can increase the plasma levels of psychiatric medications, raising the risk of side effects and toxicity. Some AEDs can alter the protein binding of psychiatric medications, affecting their free (active) concentrations. Valproate, for example, can displace other drugs from protein binding sites, increasing their free concentrations, which may enhance efficacy or toxicity (lamotrigine). Phenytoin is highly protein-bound and can interact with other highly protein-bound drugs, leading to increased free drug levels of psychiatric medications. AEDs and many psychiatric medications can cause CNS depression, leading to additive effects. Benzodiazepines and antipsychotics, when combined with AEDs, can increase sedation, respiratory depression, and cognitive impairment. SSRIs may increase the level of valproate, and tricyclic antidepressants (TCAs) can be significantly affected by inducers like carbamazepine, necessitating dosage adjustments. Concomitant use of clozapine with carbamazepine can lead to decreased clozapine levels, necessitating monitoring and possible dose adjustments. Valproate could increase level of olanzapine, leading to heightened risk of side effects [180,273,274,275,276,277,278,279,280,281,282,283,284,285,286]. Understanding the implications of these combinations is crucial, as serious adverse reactions may arise. Moreover, the effectiveness of combination therapy has been corroborated in open-label studies involving patients with both ASD and epilepsy who exhibit partial responses. These studies support combinations such as olanzapine with lithium, valproate, or carbamazepine; risperidone with lithium or valproate; and quetiapine with lithium or valproate. However, the simultaneous use of these medications must be approached with caution due to the risk of toxic symptoms at elevated doses. Additionally, acute manic episodes, extrapyramidal symptoms, physiological disturbances, or potential neurological damage could occur, affecting the brain’s structure in areas where the epilepsy focus is located [62,80,287,288,289,290,291,292,293,294,295,296,297,298,299,300].

Medical treatments for ASD and epilepsy primarily aim to manage the core symptoms of both conditions while also addressing co-occurring physical issues such as sleep disturbances, gastrointestinal problems, and sensory sensitivities. Significant challenges remain in understanding the comorbidity between ASD and epilepsy and in exploring the relationship between seizures and the central social symptoms associated with ASD. Although there is a notable prevalence of epilepsy among individuals with ASD, the precise ways in which seizures may influence social functioning in ASD are still an area of active research. Further challenges to the treatment also include the symptomatic variability of both epileptic and spectrum disorder syndromes. While some children experience recurrent seizures, others may have only a single episode in their lifetime. This inconsistency not only adds complexity to studying the relationship between seizures and ASD but also poses challenges in identifying predictive patterns and developing personalized treatment strategies [297].

## 4. Conclusions

In conclusion, the intricate relationship between ASD and epilepsy underscores the necessity for a comprehensive approach to treatment that addresses both conditions simultaneously. As research continues to evolve, it becomes increasingly clear that tailored interventions that consider the unique needs of individuals with co-occurring ASD and epilepsy can significantly enhance quality of life. Multidisciplinary strategies, including pharmacological management, which requires careful consideration of the complex interplay between medications, individual patient needs, and the potential for adverse interactions; behavioral therapies; and supportive services are crucial in optimizing outcomes for patients. Furthermore, ongoing education and training for healthcare providers are essential to ensure that they are equipped to recognize and manage the complexities of these intertwined disorders. By fostering a collaborative environment among caregivers, clinicians, and researchers, we can pave the way for innovative treatment modalities that not only mitigate symptoms but also promote overall well-being for individuals navigating the challenges of both autism and epilepsy. The journey ahead is promising, but it requires a steadfast commitment to improving understanding and support for this vulnerable population.

## Figures and Tables

**Table 1:** Diagnostic criteria of ASD.

DSM-VTR Diagnostic Criteria
A. **Persistent deficits in social communication and social interaction across multiple contexts, as manifested by the following:** **Social-emotional reciprocity:** ○ Abnormal social approach and failure of normal back-and-forth conversation. ○ Reduced sharing of interests, emotions, or affect. ○ Failure to respond to social interactions. **Nonverbal communicative behaviors:** ○ Abnormalities in eye contact and body language or deficits in understanding and use of gestures. ○ Total lack of facial expressions and nonverbal communication. **Developing, maintaining, and understanding relationships:** ○ Difficulties adjusting behavior to suit various social contexts. ○ Difficulties in sharing imaginative play or in making friends. ○ Absence of interest in peers.
B. **Restricted, repetitive patterns of behavior, interests, or activities, as manifested by at least two of the following:** Stereotyped or repetitive motor movements, use of objects, or speech: ○ Simple motor stereotypies, lining up toys, or flipping objects. ○ Echolalia, idiosyncratic phrases. **Insistence on sameness, inflexible adherence to routines, or ritualized patterns of behavior:** ○ Extreme distress at small changes. ○ Difficulty with transitions. ○ Rigid thinking patterns. **Highly restricted, fixated interests that are abnormal in intensity or focus:** ○ Strong attachment to or preoccupation with unusual objects. ○ Excessively focused interests. **Hyper- or hyporeactivity to sensory input or unusual interest in sensory aspects of the environment:** ○ Indifference to pain/temperature. ○ Adverse response to specific sounds or textures. ○ Excessive smelling or touching of objects.
C. **Symptoms must be present in the early developmental period (typically recognized in the first two years of life), but may not become fully manifest until social demands exceed limited capacities, or may be masked by learned strategies in later life.**
D. **Symptoms cause clinically significant impairment in social, occupational, or other important areas of current functioning.**
E. **These disturbances are not better explained by intellectual disability (intellectual developmental disorder) or global developmental delay. Intellectual disability and ASD frequently co-occur; to make comorbid diagnoses of ASD and intellectual disability, the social communication criteria must be met without the need for the performance on standardized tests to be below the intellectual disability threshold.**
**Specifiers:** Current severity (Level 1, Level 2, Level 3). With or without accompanying intellectual impairment. With or without accompanying language impairment. Associated with a known medical or genetic condition or environmental factor. Associated with another neurodevelopmental, mental, or behavioral disorder.

**Table 2:** Pharmacological treatment ASD.

Pharmacological Treatment ASD
Pharmacological treatments can help manage specific symptoms associated with the disorder. Treatment strategies often focus on addressing co-occurring conditions, such as anxiety, depression, attention-deficit/hyperactivity disorder (ADHD), and irritability.
**(1)** **Irritability and aggression:** **antipsychotics** ○ I line of treatment ▪ aripiprazole ▪ risperidone ○ II line of treatment ▪ quetiapine, ▪ ziprasidone, ▪ paliperidone, ▪ haloperidol ▪ chlorpromazine ▪ Clozapine (disruptive behaviors) **mood stabilizers** ○ Valproate **(2)** **Co-occurrence of ADHD:** **methylphenidate** **alpha-2 adrenergic agonists:** ○ Clonidine ○ guanfacine **(3)** **stereotypies:** **antipsychotics:** ○ risperidone **xanthine derivatives:** ○ pentoxifylline. **(4)** **Anxiety and depression:** **antidepressants** ○ **Selective Serotonin Reuptake Inhibitors (SSRIs):** ▪ Citalopram ▪ Fluoxetine, ▪ Sertraline **anxiolytics** **noradrenergic and specific serotonergic antidepressants (NaSSA):** ○ mirtazapine ○ buspirone **II line of treatment: Methylphenidate and risperidone** **(5)** **Sleep disorder:** **Benzodiazepines:** ○ clonazepam **Melatonin** **alpha-2 adrenergic agonist:** ○ clonidine **(6)** **OCD:** **SSRIs:** ○ fluoxetine ○ fluvoxamine ○ citalopram ○ sertraline ○ escitalopram ○ clomipramine **antipsychotics.** **(7)** **Seizures and repetitive behaviors** **Antiepileptic drugs:** ○ valproate **(8)** **Schizophrenia:** **antipsychotics**

**Table 3:** Anti-epileptic drugs.

Classification of Antiepileptic Drugs
AEDs Can Be Classified into:
**Based on Mechanism of Action:**
Sodium Channel Blockers ○ Phenytoin ○ Carbamazepine ○ Lamotrigine ○ Lacosamide ○ Rufinamide Calcium Channel Modulators ○ Ethosuximide (T-type calcium channel blocker) ○ Gabapentin (modulates voltage-gated calcium channels) ○ Pregabalin (modulates voltage-gated calcium channels) GABAergic Drugs ○ Benzodiazepines (e.g., Diazepam, Lorazepam) ○ Phenobarbital ○ Tiagabine (inhibits GABA reuptake) ○ Vigabatrin (inhibits GABA transaminase) Glutamate Receptor Antagonists ○ Topiramate (also has other mechanisms) ○ Perampanel (AMPA receptor antagonist) Other Mechanisms ○ Levetiracetam (modulates synaptic vesicle protein 2A) ○ Zonisamide (multiple mechanisms, including sodium and calcium channel modulation) ○ Felbamate (NMDA receptor antagonist and enhances GABA activity)
**Based on Chemical Structure:**
Hydantoins ○ Phenytoin Carboxamides ○ Carbamazepine ○ Oxcarbazepine ○ Eslicarbazepine Succinimides ○ Ethosuximide Benzodiazepines ○ Diazepam ○ Clonazepam ○ Lorazepam Barbiturates ○ Phenobarbital Amines ○ Gabapentin ○ Pregabalin Sulfonamides ○ Zonisamide Other Structures ○ Levetiracetam (pyrrolidine) ○ Topiramate (sugar-derived compound) ○ Lacosamide (vinylsultam derivative)
**Based on Seizure Type**
Generalized Seizures ○ Valproate ○ Lamotrigine ○ Levetiracetam ○ Topiramate ○ Zonisamide ○ Benzodiazepines Focal Seizures ○ Carbamazepine ○ Phenytoin ○ Oxcarbazepine ○ Lacosamide ○ Gabapentin ○ Pregabalin Absence Seizures ○ Ethosuximide ○ Valproate ○ Lamotrigine
**Newer AEDs:**
Lacosamide Perampanel Eslicarbazepine Brivaracetam Cenobamate

## Data Availability

The datasets used and analyzed during the current study are available from the corresponding author on reasonable request.

## Abbreviations

a-CGH	array comparative genomic hybridization
ASD	autism spectrum disorder
MRI	magnetic resonance imaging
MDT	multi-disciplinary team
EEG	electroencephalogram
ADOS	Autism Diagnostic Observation Schedule
ADI-R	Autism Diagnostic Interview-Revised (ADI-R)
DSM 5TR	Diagnostic and Statistical Manual of Mental Disorders, Fifth Edition, Text Revision
MSEL	Mullen Scales of Early Learning (MSEL)
CDI	MacArthur–Bates Communicative Developmental Inventory
WHO	World Health Organization
CDC	Centers for Disease Control and Prevention (CDC)
mTOR	mammalian target of rapamycin
MECP2	methyl-CpG-binding protein 2
CNS	central nervous system (CNS)
GI	gastrointestinal tract
SITBS	self-injurious thoughts and behaviors
C-SSRS	Columbia-Suicide Severity Rating Scale
ADHD	attention-deficit/hyperactivity disorder
IDD	intellectual developmental disorder
IQ	intelligence quotient
PI3K	phosphoinositide 3-kinases
NRXN	neurexins
NLGN	neuroligin
OCD	obsessive-compulsive disorder
FXS	Fragile X syndrome
DS	Down syndrome
NF1	neurofibromatosis type I
TSC	tuberous sclerosis complex
CPK	creatine phosphokinase
ED	emergency department
SIB	self-injurious behavior
NaSSA	noradrenergic and specific serotonergic antidepressants
SSRIs	selective serotonin reuptake inhibitors
ILAE	International League Against Epilepsy (ILAE)
GGE	genetic generalized epilepsy
FE	focal epilepsy (FE)
FMTLE	mesial temporal lobe epilepsy
ADLTE	autosomal dominant lateral temporal epilepsy
ADNFLE	autosomal dominant nocturnal frontal lobe epilepsy
HS	hippocampal sclerosis
FS	febrile seizures
LGI1	leucine-rich glioma-inactivated 1 (LGI1)
nAChR	neuronal nicotinic acetylcholine receptor
LMICs	middle-income countries
HIV	human immunodeficiency virus
BBB	Blood–brain barrier
GABA	gamma-aminobutyric acid, γ-aminobutyric acid
NMDA-R	N-methyl-D-aspartate receptor
AMPA	α-amino-3-hydroxy-5-methyl-4-isoxazolepropionic acid
mGLuR5	metabotropic glutamate receptor 5 (mGluR5),
GAD65	glutamic acid decarboxylase 65 (GAD65),
ANNA-1	antineuronal nuclear antibody type 1 (ANNA-1),
IL-1R/TLR	IL-1 receptor/toll-like receptor
PAMP	pathogen-associated molecular patterns
DAMPs	damage-associated molecular patterns
COX2	cyclooxygenase-2
TGF-β/Smad	transforming growth factor-β/small mothers against decapentaplegic
HMGB1	high-mobility group box-1 (HMGB1)
SUDEP	sudden death in wpilepsy
SCN1A	sodium voltage-gated channel alpha subunit 1
GABA	4-aminobutanoic acid
CT	computed tomography
SPECT	single-photon emission computed tomography
SIADH	syndrome of inappropriate secretion of antidiuretic hormone
CBZ	carbamazepine
CRMP2	collapsing response mediator protein 2
SV2A	synaptic vesicle glycoprotein 2A
AED	antiepileptic drugs
RTT	Rett syndrome
UBE3A	ubiquitin-protein ligase E3A (UBE3A,
ACC	agenesis of the corpus callosum
SHANk3	multiple ankyrin repeat domains 3
ARX	aristaless-related homeobox
FOXP2	forkhead box P2
SCN1A	sodium voltage-gated channel alpha aubunit 1
SCN2A	sodium voltage-gated channel alpha subunit 2
SCN8A	sodium voltage-gated channel alpha subunit 8
KCNB1	potassium voltage-gated channel subfamily B member 1
CACNA1A	calcium voltage-gated channel subunit alpha1 A
SHANK3	SH3 and multiple ankyrin repeat domains 3
CDKL5	cyclin-dependent kinase-like 5
STXBP1	syntaxin binding protein 1
NRXN1	neurexin 1
PCDH19	protocadherin 19
CASK	calcium/calmodulin-dependent serine protein kinase
SYNGAP1	synaptic Ras GTPase activating protein 1
YWHAG	tyrosine 3-monooxygenase/tryptophan 5-monooxygenase activation protein gamma
PACS2	hosphofurin acidic cluster sorting protein 2
ACTL6B	actin like 6B
MEF2C	myocyte enhancer factor 2C
FOXG1	forkhead box G1
VAP	valproic acid
TCAs	tricyclic antidepressants (TCAs)
EKG	electrocardiogram
KS	Klinefelter syndrome
ADEM	acute disseminated encephalomyelitis
